# Genomes of cryptic chimpanzee *Plasmodium* species reveal key evolutionary events leading to human malaria

**DOI:** 10.1038/ncomms11078

**Published:** 2016-03-22

**Authors:** Sesh A. Sundararaman, Lindsey J. Plenderleith, Weimin Liu, Dorothy E. Loy, Gerald H. Learn, Yingying Li, Katharina S. Shaw, Ahidjo Ayouba, Martine Peeters, Sheri Speede, George M. Shaw, Frederic D. Bushman, Dustin Brisson, Julian C. Rayner, Paul M. Sharp, Beatrice H. Hahn

**Affiliations:** 1 Department of Medicine, University of Pennsylvania, Philadelphia, 19104 Pennsylvania, USA; 2 Department of Microbiology, University of Pennsylvania, Philadelphia, 19104 Pennsylvania, USA; 3 Institute of Evolutionary Biology and Centre for Immunity, Infection and Evolution, University of Edinburgh, EH9 3FL Edinburgh, UK; 4 Unité Mixte Internationale 233, Institut de Recherche pour le Développement (IRD), INSERM U1175, University of Montpellier, 34394 Montpellier, France; 5 Sanaga-Yong Chimpanzee Rescue Center, IDA-Africa, Portland, 97204 Oregon, USA; 6 Department of Biology, University of Pennsylvania, Philadelphia, 19104 Pennsylvania, USA; 7 Malaria Programme, Wellcome Trust Sanger Institute, Wellcome Genome Campus, CB10 1SA Cambridge, UK

## Abstract

African apes harbour at least six *Plasmodium* species of the subgenus *Laverania*, one of which gave rise to human *Plasmodium falciparum*. Here we use a selective amplification strategy to sequence the genome of chimpanzee parasites classified as *Plasmodium reichenowi* and *Plasmodium gaboni* based on the subgenomic fragments. Genome-wide analyses show that these parasites indeed represent distinct species, with no evidence of cross-species mating. Both *P. reichenowi* and *P. gaboni* are 10-fold more diverse than *P. falciparum*, indicating a very recent origin of the human parasite. We also find a remarkable *Laverania*-specific expansion of a multigene family involved in erythrocyte remodelling, and show that a short region on chromosome 4, which encodes two essential invasion genes, was horizontally transferred into a recent *P. falciparum* ancestor. Our results validate the selective amplification strategy for characterizing cryptic pathogen species, and reveal evolutionary events that likely predisposed the precursor of *P. falciparum* to colonize humans.

## Abstract (web summary)

African apes harbour six *Plasmodium* species, one of which gave rise to the human malaria parasite. Here, Sundaraman *et al*. use selective whole-genome amplification to determine genome sequences from two chimpanzee *Plasmodium* species, shedding light on the evolutionary origin of the human parasite.

## Graphical abstract

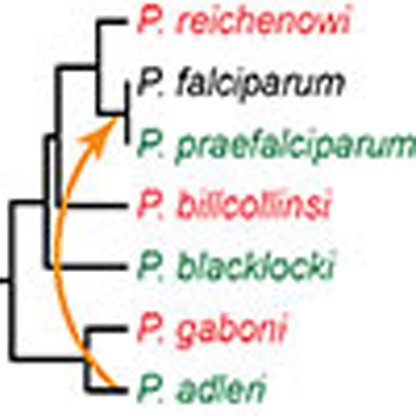

P*lasmodium falciparum*, the cause of malignant malaria in humans, is only distantly related to other human malaria parasites and has been classified into a separate subgenus, termed *Laverania*^1^. Both chimpanzees and gorillas have long been known to harbour parasites morphologically indistinguishable from *P. falciparum*, but only one ape *Laverania* species, *P. reichenowi* from chimpanzees, has been described^1^. *P. falciparum* and *P. reichenowi* are closely related, which led to the hypothesis that they had co-diverged with their hosts, since the human–chimpanzee common ancestor 5–10 million years ago^2^. However, we and others have obtained evidence that points to the existence of additional *Plasmodium* species naturally infecting apes^3^,^4^,^5^,^6^,^7^. Phylogenetic analyses of faecal-derived mitochondrial, apicoplast and nuclear DNA sequences demonstrated that wild-living chimpanzees (*Pan troglodytes*) and western gorillas (*Gorilla gorilla*) are each infected with at least three divergent, host-specific, parasite lineages that appear to represent distinct *Laverania* species^7^. Moreover, one of the newly identified species from western gorillas is very closely related to *P. falciparum*. In phylogenetic trees of full-length mitochondrial DNA (mtDNA) sequences, all extant strains of *P. falciparum* form a monophyletic clade within the radiation of this gorilla parasite, indicating that *P. falciparum* originated following a relatively recent jump from gorillas to humans^7^. *P. falciparum* differs from other human malaria parasites in many aspects of its biology, in particular its virulence^8^. Thus, characterizing its ape *Laverania* relatives provides an opportunity to understand the pathogenicity of this parasite and the reasons for its emergence.

With the exception of *P. reichenowi*, none of the other ape *Laverania* species have been formally identified. In one study, *falciparum*-like ring stages were observed in chimpanzee blood samples, and divergent mtDNA sequences were obtained that were classified as a new species, *P. gaboni*^3^. A third species of chimpanzee parasites, termed *P. billcollinsi*, has been identified solely on the basis of DNA sequences, albeit from mitochondrial, apicoplast as well as nuclear genomes^7^,^9^,^10^. Similarly, the three distinct gorilla parasite species, *P. praefalciparum* (the precursor of *P. falciparum*), *P. blacklocki* and *P. adleri*, have not been cultured or visually characterized, but are clearly defined based on DNA sequence data^7^,^9^,^10^. Given the similarity of *P. gaboni* to *P. falciparum* (and thus to *P. reichenowi*) in microscopic studies, it seems likely that all of these ape *Laverania* parasites represent cryptic (morphologically indistinguishable) species. Because of the endangered status of African apes that prohibits blood collection from wild populations, and ethical concerns that preclude experimental infection of captive individuals, it has not been possible to obtain the morphological and biological data required for classical taxonomic approaches. Nonetheless, existing genetic data indicate that ape *Laverania* parasites fall into six distinct clades, each at least as divergent from one another as *P. falciparum* is from *P. reichenowi*^9^. Moreover, the strict host specificity of these parasites, as well as their prevalence and distribution throughout the range of their natural host species, argue strongly that the ape *Laverania* species are actively transmitted and cause productive infections.

Many apes appear to be simultaneously co-infected by more than one *Laverania* species^7^. Elucidation of the mechanisms that prevent these parasites from interbreeding, as well as the basis of their host specificity, is of obvious interest. One approach to examine whether the *Laverania* species are truly isolated, and to understand their evolutionary history and host tropism, would be to obtain their genome sequences. However, this has been hampered by a lack of ape blood samples containing high levels of parasites and the fact that none of these parasite species can be cultured. All *Plasmodium* reference genomes generated to date were derived from purified parasites that were grown to high titres in red blood cells (RBCs) *in vitro* or in susceptible host species *in vivo*^11^,^12^,^13^,^14^,^15^. Since RBCs from chimpanzees and gorillas are not readily accessible, efforts to propagate ape *Laverania* parasites *in vitro* have remained unsuccessful. To date, only a single-genome sequence of the chimpanzee parasite *P. reichenowi* has been determined, following extensive *in vivo* passage and amplification in experimentally infected, splenectomised chimpanzees^12^,^16^. However, this method of parasite enrichment is no longer considered ethical.

In this study, we apply a strategy that permits the selective amplification of near-full-length *Plasmodium* genomes from ape blood with microscopically undetectable parasitemia. Using this approach, we obtain genome sequences from three chimpanzee parasites, one classified as *P. reichenowi* and two as *P. gaboni* based on short PCR fragments. The genome-wide analyses provide new insights into the evolutionary history of *P. falciparum*. Remarkably, each of the two chimpanzee *Laverania* species exhibits about 10 times more within-species genetic diversity than is found among a global sample of *P. falciparum*, indicating that the human parasite has been through a severe genetic bottleneck, consistent with a very recent origin. We also describe the rapid expansion and diversification of a multigene family (FIKK) that is unique to the *Laverania* subgenus, as well as a horizontal transfer of a short (8 kb) chromosomal region that encodes two functionally related, essential invasion genes. Thus, we validate a new approach for characterizing cryptic pathogen species, and reveal adaptive processes that may have allowed the gorilla precursor of *P. falciparum* to cross the species barrier to humans.

## Results

### Selective whole-genome amplification of *Laverania* parasites

Traditional whole-genome amplification methods utilize the highly processive phi29 polymerase and random primers to generate DNA fragments of up to 70 kb in length, but amplify all templates within a sample with near-uniformity^17^,^18^. Since microbial and host genomes differ in the frequency of common sequence motifs, we reasoned that it should be possible to design primers that would amplify pathogens selectively, even if they represented only a small fraction of the sample DNA. Testing this concept on *Wolbachia*-infected fruit flies, we found that selective whole-genome amplification (SWGA) generated sufficient quantities of bacterial genomes for next-generation sequencing^19^. To extend this method to more complex eukaryotic pathogens, here we tested whether SWGA could amplify the multi-chromosomal genomes of *Plasmodium* parasites from unprocessed human and ape blood samples.

**Figure 1:**
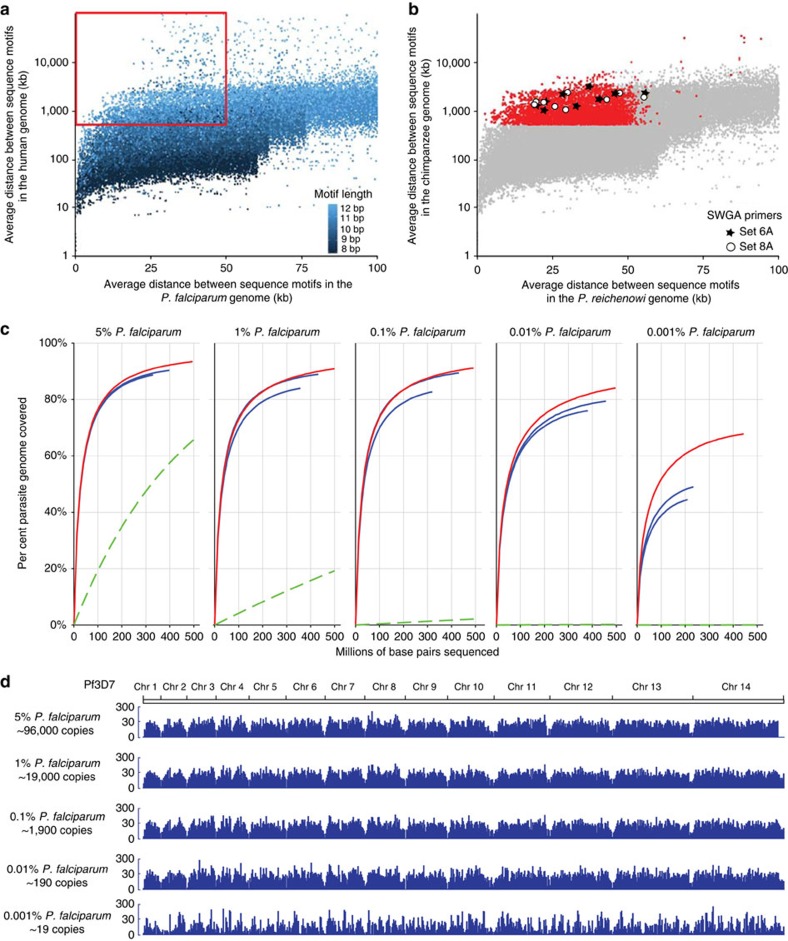
SWGA of *Plasmodium* parasites. (**a**,**b**) Selection of SWGA primer sets. (**a**) The average distance (kb) between the 10,000 most frequent parasite motifs (colour coded by length) is plotted for both the *P. falciparum* (Pf3D7) and human (GRCh37) genomes. The red box highlights motifs that are spaced (on average) <50,000 bp apart in the *P. falciparum*, but >500,000 bp apart in the human genome. (**b**) Average distances between the sequence motifs shown in **a**, but plotted for the *P. reichenowi* (PrCDC) and chimpanzee (Pan_troglodytes-2.1.4) genomes. Red dots indicate all motifs that fall within the red box in **a**, with circles and stars denoting those selected for SWGA primer sets 6A and 8A, respectively (Supplementary Fig. 1). (**c**) Validation of the SWGA primer sets. Human genomic DNA spiked with known quantities of *P. falciparum* DNA (5–0.001%) was subjected to consecutive rounds of SWGA, using primer set 6A in the first and primer set 8A in the second round. The number of total base pairs (in millions) sequenced is shown in relation to the per cent coverage of the *P. falciparum* (Pf3D7) genome for five parasite concentrations. DNA mixtures were subjected to two independent amplifications, with individual and combined results shown in blue and red, respectively (the expected genome coverage without SWGA is shown in green). (**d**) MiSeq read depth for all 14 chromosomes across the Pf3D7 genome shown for one representative amplification at each of five parasite concentrations (individual chromosomes are drawn to scale as indicated on top). For each parasite/human DNA mixture, the percentage of *P. falciparum* and estimated number of genome copies are indicated. An expanded view of coverage across chromosome 9 is shown in Supplementary Fig. 2.

To identify primers that would selectively bind to *Laverania* DNA, we determined the frequency of short sequence motifs (8–12 bp in length) in both the *P. falciparum* and human genomes (Supplementary Fig. 1). This approach identified 2,418 motifs that occurred frequently (that is, were spaced on average <50,000 bp apart) in the *P. falciparum* genome, but only rarely in the human genome (that is, were spaced on average >500,000 bp apart; Fig. 1a). These same motifs were also over-represented in *P. reichenowi* relative to the chimpanzee genome, suggesting that they could be used to selectively amplify other *Laverania* parasite species (Fig. 1b). To identify the best possible primers, we first filtered the 2,418 motifs based on their DNA-binding properties and then selected two primer sets based on their ability to bind evenly across the *P. falciparum* genome (Supplementary Fig. 1).

To validate the SWGA primer sets, we tested them using human DNA samples spiked with known quantities (0.001–5%) of *P. falciparum* DNA. These experiments showed that SWGA amplified *P. falciparum* genomes with remarkable breadth and selectivity over a wide range of concentrations, especially when results from independent amplifications were combined (Fig. 1c). Of ∼2.5 million MiSeq reads derived from samples containing as little as 0.001% *P. falciparum* DNA (19 genome equivalents), ∼1.7 million (70%) mapped to the *P. falciparum* genome, indicating a 70,000-fold enrichment of the parasite DNA compared with that of the host (Table 1). Read coverage was even across all 14 chromosomes, except for the sub-telomeres where low sequence complexity precluded accurate mapping (Fig. 1d). Amplification of the lowest (0.001%) parasite concentration was more stochastic (Fig. 1d and Supplementary Fig. 2). However, since combining multiple SWGA replicates improved coverage even at this low dilution, it is likely that the stochastic coverage is the result of amplification of a very limited number of starting genomes (19 genomic equivalents). Thus, SWGA generated high-quality *Plasmodium* core genomes from samples containing large quantities of contaminating host DNA.

### Sequence analysis of *P. gaboni* and *P. reichenowi* genomes

We next used SWGA to amplify the genomes of three chimpanzee parasites, representing both close (*P. reichenowi*) and very distant (*P. gaboni*) relatives of *P. falciparum*^9^. Whole-blood samples were obtained from (blood smear negative) sanctuary chimpanzees during their annual health examination and tested for *Plasmodium* infection using conventional PCR. Parasite DNA-positive blood samples were further characterized by limiting dilution (single template) PCR amplification of eight mitochondrial, apicoplast and nuclear loci to determine their *Plasmodium* species composition^7^,^10^. This analysis identified one sample (SY57) to contain almost exclusively (>99%) *P. reichenowi* DNA and two others (SY75 and SY37) to contain only *P. gaboni* DNA (Supplementary Table 1). In each case, the parasites contributed only a miniscule fraction of the total blood DNA (0.0054, 0.14 and 0.00081% for SY57, SY75 and SY37, respectively). To reduce the contaminating host DNA, we digested all samples with methylation-dependent restriction enzymes (MspJI and FspEI) known to cleave ape, but not *Plasmodium*, genomic DNA^20^, and then used the digestion products for SWGA and Illumina sequencing. This approach yielded 27, 31 and 39 million MiSeq reads for samples SY57, SY75 and SY37, respectively, of which 89, 73 and 61%, respectively, mapped to *Plasmodium* sequences (Supplementary Table 1). Sequence coverage was even across all 14 chromosomes, including near the ends of some chromosomes, with no evidence for selective sequence loss (Supplementary Fig. 3). Reads from sample SY57 were mapped to the *P. reichenowi* PrCDC reference sequence and shown to cover 96% of its genome (at a fivefold or higher read coverage). Since there is no published *P. gaboni* genome, reads from samples SY75 and SY37 were mapped to the *P. falciparum* Pf3D7 reference sequence and shown to cover 79 and 69% of its genome (at ≥5 ×), respectively (Supplementary Table 1). This lower coverage was not due to a reduction in selective amplification, but instead the result of the difficulty of mapping reads to a highly divergent reference sequence.

Using reference-guided iterative assembly^21^, we generated draft genomes for PrSY57 and PgSY75, which contained 18.9 and 18.5 Mb of chromosomal, as well as 0.8 and 1.5 Mb of unplaced (bin) sequences, respectively (Table 2). Due to the very small quantities of parasite DNA, sequence coverage for PgSY37 was lower, yielding 15 Mb of chromosomal and 9 Mb of unplaced sequences. Syntenic annotation transfer and *ab initio* gene prediction identified a total of 4,920, 4,962 and 4,179 full-length and partial protein-coding genes in PrSY57, PgSY75 and PgSY37, respectively, which included 98.3, 98.7 and 85.7%, respectively, of the core genes in the respective reference sequences (Table 2). In genomic regions that were syntenic among all three species, there were only four intact *P. gaboni* genes that were missing in *P. falciparum* and/or *P. reichenowi*, and only three intact *P. reichenowi* and/or *P. falciparum* genes that were absent from *P. gaboni* (Supplementary Table 2, Supplementary Fig. 4). Of 76 pseudogenes examined in the three *Laverania* species, only 7, 13 and 10 were specific to *P. falciparum, P. reichenowi* and *P. gaboni*, respectively (Supplementary Table 3). Given the evolutionary relationships among these species^9^, this indicates that the core genome of ape and human parasites is highly conserved, even among the most divergent members of the *Laverania* subgenus.

### Within-species diversity in *P. reichenowi* and *P. gaboni*

Availability of new *P. reichenowi* and *P. gaboni* genomes allowed us to examine the within-species diversity among chimpanzee parasites. For comparison, the intra-species diversity of *P. falciparum* was calculated using published SNP data from 12 geographically diverse field isolates (see the ‘Methods' section for details). Comparing more than 3,000 genes, we found that the two *P. gaboni* genomes (PgSY75 and PgSY37) differed at 0.4% of all coding sites, and 1.1% of fourfold degenerate (silent) sites. Similarly, the two *P. reichenowi* genomes (PrSY57 and PrCDC) differed at 0.3% of all coding sites, and 0.9% of fourfold degenerate sites (Table 3). Note that, for every gene, the divergence between the two *P. gaboni* sequences, or between the two *P. reichenowi* sequences, was lower than between these two species, consistent with the premise that *P. gaboni* and *P. reichenowi* are genetically isolated.

**Figure 2:**
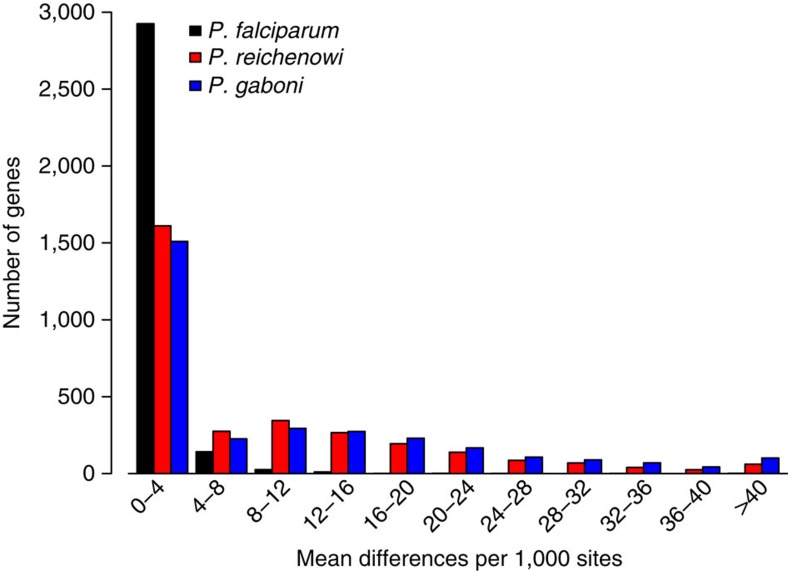
Sequence diversity within three *Laverania* species. The average pairwise nucleotide sequence diversity is shown for 3,111 orthologous core genes at fourfold degenerate sites for 12 geographically diverse strains of *P. falciparum* (black), two strains of *P. reichenowi* (red) and two strains of *P. gaboni* (blue). For *P. falciparum* field isolates, diversity information was obtained from SNP data (only data sets representing single-parasite strains were used for analysis; see the ‘Methods' section for detail).

In contrast, 12 field isolates of *P. falciparum* selected from countries around the world differed on average at only 0.04% of all coding sites, and 0.08% of fourfold degenerate sites (Table 3). To ensure that the higher diversity among the ape parasites was not an artefact of the SWGA method, we amplified several nuclear loci that exhibited particularly high sequence diversity (three from *P. gaboni* and four from *P. reichenowi*) using limiting dilution PCR (Supplementary Table 4). The resulting sequences were identical to the SWGA-derived genomes except for two indels in difficult-to-assemble regions, which had been excluded from the diversity calculations, thus further validating the accuracy of the SWGA method (Supplementary Fig. 5). The distributions of diversity levels across genes were very similar in *P. reichenowi* and *P. gaboni* (Fig. 2). This was also the case when the within-species diversity for *P. reichenowi* or *P. gaboni* was compared with the maximum pairwise divergence obtained for each gene among the 12 *P. falciparum* field isolates (Supplementary Fig. 6). In all comparisons, the difference between ape and human parasites reflected higher diversity levels in genes distributed across the entire core genome. Thus, for both chimpanzee parasite species, including two *P. gaboni* strains from the same location, the genetic diversity is about ten times higher than that seen among *P. falciparum* strains from different geographic regions across the globe. This reduced diversity in *P. falciparum* is consistent with a severe population bottleneck, which most likely occurred at the cross-species transmission from gorilla to human.

### *Laverania*-specific expansion of the FIKK multigene family

**Figure 3:**
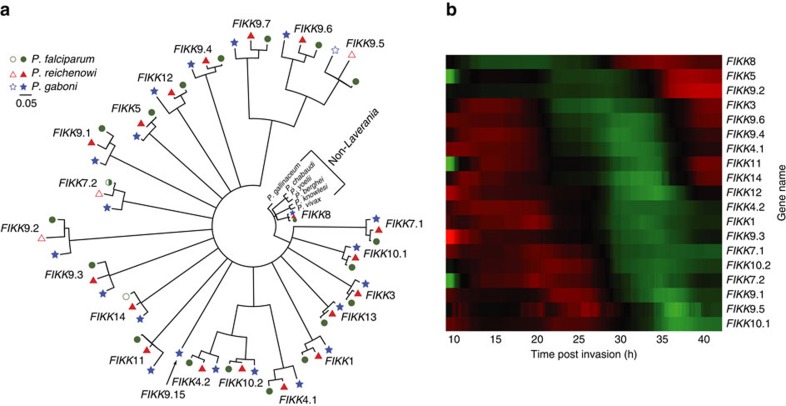
Expansion and diversification of the *FIKK* multigene family in the *Laverania* subgenus. (**a**) Phylogeny of *FIKK* genes from *P. falciparum* (green), *P. reichenowi* (red), *P. gaboni* (blue) and non-*Laverania* species (black). *FIKK* genes are labelled according to their *P. falciparum* orthologues, with the new *P. gaboni* gene designated *FIKK*9.15. Open symbols indicate pseudogenes in all members of the species (*FIKK*7.2 is intact in some strains of *P. falciparum*). The tree was inferred using maximum likelihood methods^69^ using an alignment of first and second codon positions. Internal branches with bootstrap support of <70% are collapsed. Scale bar, 0.05 substitutions per site. (**b**) Expression profiles of *P. falciparum FIKK* genes. Previously published microarray data^23^ from 21 clonal *P. falciparum* strains were used to calculate the expression levels of 19 FIKK genes at different time points during the intra-erythrocytic lifecycle (data for the remaining *FIKK* gene were not available). Colours represent mRNA expression levels relative to a reference pool, with red, black and green indicating higher, equal and lower expression levels than the reference pool, respectively. Genes were arranged to illustrate the sequential nature of *FIKK* gene expression.

To gain insight into the host specificity of *Laverania* parasites, we examined members of multigene families known to function at the host-parasite interface. Many of these, including members of the *var*, *rif* and *stevor* families, could not be completely assembled because of their extreme variability and subtelomeric location, although *var*-like genes have been shown to be present in all ape *Laverania* species^10^. One family of putative protein kinases, termed FIKK (after a conserved Phe-Ile-Lys-Lys motif in their amino acid sequence), was of particular interest because it expanded from a single gene, present in other *Plasmodium* species, to 21 genes in both *P. falciparum* and *P. reichenowi*^12^,^22^. Remarkably, the new *P. gaboni* genome contained 22 *FIKK* genes, 21 of which represented clear syntenic orthologues of corresponding *P. falciparum* and *P. reichenowi* genes as demonstrated by phylogenetic analysis (Fig. 3a) and chromosomal location (Supplementary Table 5). The remaining *P. gaboni* gene on chromosome 9, termed *FIKK*9.15, did not have an orthologue in *P. falciparum* and *P. reichenowi.* This gene originated before the last common ancestor of the extant *Laverania* (Fig. 3a), but appears to have been lost in a common ancestor of *P. falciparum* and *P. reichenowi* (Supplementary Fig. 7a). The closest relative of *P. gaboni*, the gorilla parasite *P. adleri*, retained a *FIKK*9.15 orthologue (Supplementary Fig. 7b). These data indicate that the FIKK gene family underwent an unprecedented burst of gene duplications and rapid diversification very early in *Laverania* evolution, followed by a period of greatly reduced divergence rates and near stasis of gene copy numbers after the radiation of extant *Laverania* species (Fig. 3a).

Although their exact functions remain to be determined, the *P. falciparum FIKK* genes are expressed at different time points during the erythrocytic cycle^23^ (Fig. 3b), with all but the ancestral *FIKK8* believed to be exported into the host erythrocyte to contribute to the remodelling of its cytoskeleton and surface membrane structures^22^,^24^,^25^ The slow rate of evolution of *FIKK8* (Fig. 3a) suggests that it has retained its original cytosolic function, consistent with the very similar expression profiles of *FIKK8* orthologues in *P. falciparum* and *P. vivax*^23^,^26^. In contrast, all other family members appear to have acquired novel, non-redundant and seemingly essential functions, since only very few have become pseudogenes in one or more *Laverania* species. For example, *FIKK14* is a pseudogene in *P. falciparum* (Fig. 3a), but the gene appears functional in *P. praefalciparum*, as well as *P. reichenowi*, *P. gaboni*, and *P. adleri*, indicating that the inactivating mutation occurred very recently (Supplementary Fig. 8a). Similarly, *FIKK7.2* is a pseudogene in *P. falciparum* (Fig. 3a), but is intact in *P. praefalciparum*, as well as *P. gaboni* and *P. adleri*, although it has undergone independent inactivation in *P. reichenowi* (Supplementary Fig. 8b). In contrast, *FIKK9.5* is a pseudogene in both *P. gaboni* and *P. reichenowi*, but is intact in *P. falciparum*, indicating that it also must have retained its function throughout much of the diversification of the *Laverania* subgenus (Fig. 3a).

### Horizontal transfer of two essential invasion genes

**Figure 4:**
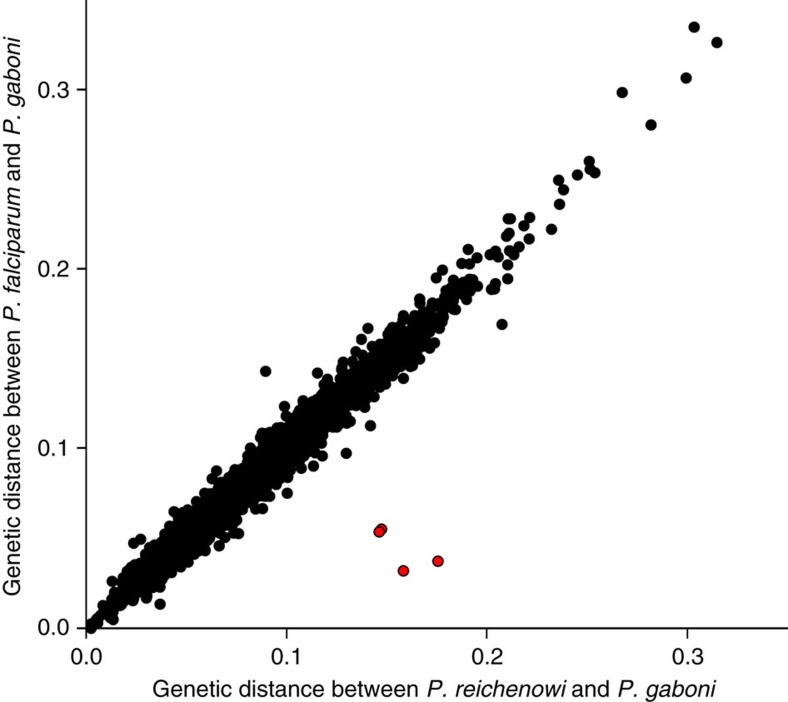
Genome-wide comparison of *Laverania* inter-species distances. Pairwise inter-species distances (substitutions per site) were calculated for all genes (dots) for which orthologs were identified in all three *Laverania* species (*n*=4,500), and the *P. reichenowi*–*P. gaboni* (*x*-axis) and *P. falciparum*–*P. gaboni* (*y*-axis) distances were plotted. Four genes that exhibit an unusually high *P. reichenowi*–*P. gaboni* and an unusually low *P. falciparum*–*P. gaboni* distance are highlighted in red.

**Figure 5:**
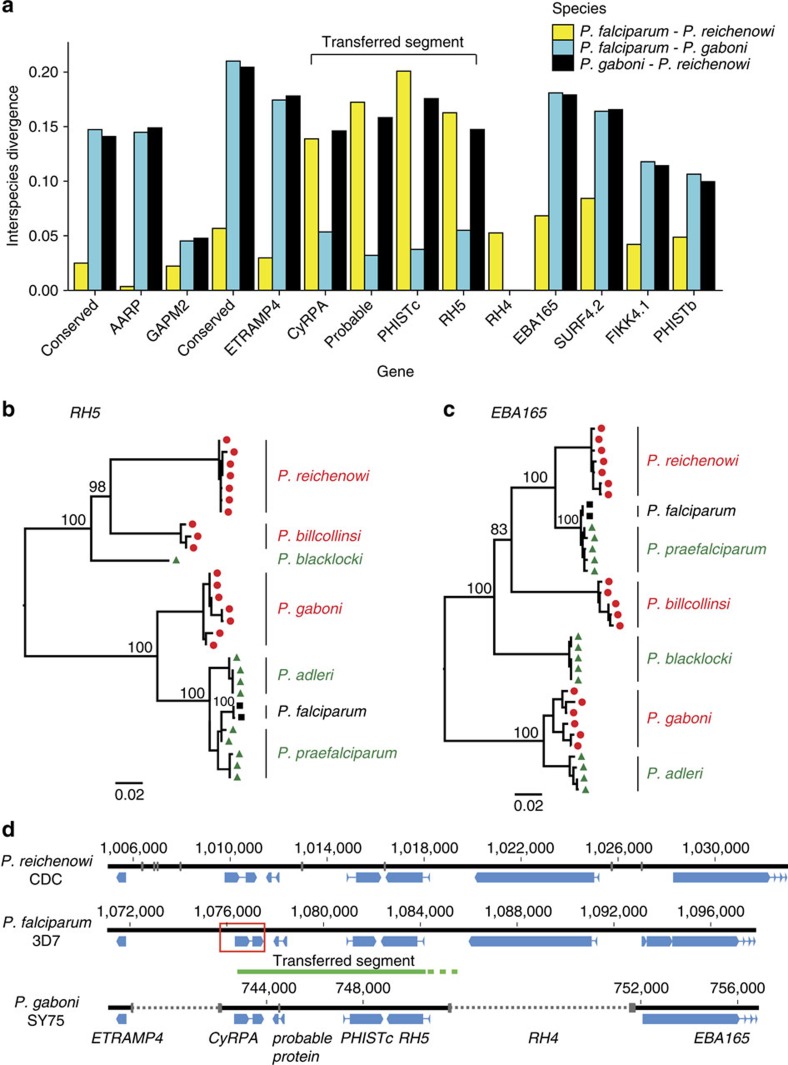
Horizontal transfer of two essential invasion genes. (**a**) Identification of an 8 kb transferred segment on chromosome 4. Inter-species distances (colour coded) are shown for syntenic orthologs of *P. falciparum*, *P. reichenowi* and *P. gaboni*. Four genes, including the essential invasion genes *CyRPA* and *RH5*, exhibit an unusually high *P. falciparum*–*P. reichenowi* (yellow) and an unusually low *P. falciparum*–*P. gaboni* (aqua) distance, respectively. Genes are ordered by chromosomal location. Since *RH4* is absent from *P. gaboni* (see Supplementary Fig. 4), only the *P. falciparum*–*P. reichenowi* distance is shown. (**b**,**c**) Phylogenetic relationships of *Laverania RH5* and *EBA165* sequences, revealing an unexpectedly close relationship between the *P. praefalciparum/P. falciparum* lineage and the *P. adleri* lineage in *RH5*. *Laverania* parasites are colour coded according to their host species (chimpanzee, red; gorilla, green; human, black). Trees were inferred by maximum likelihood methods^69^. Numbers at internal nodes represent bootstrap support values (only numbers >80% are shown). Scale bar, 0.02 substitutions per site (additional phylogenies are shown in Supplementary Fig. 9). (**d**) Schematic diagram of the horizontal transfer region on chromosome 4 in *P. reichenowi* CDC (top), *P. falciparum* 3D7 (middle) and *P. gaboni* SY75 (bottom). Genes are shown in blue; grey bars and broken lines indicate genome gaps (numbering is relative to the first base of chromosome 4). A green line indicates the location of the transferred segment in *P. falciparum*, with the broken part indicating that the 3′-break point is unclear. The region surrounding the 5′-break point is highlighted by a red box and shown in greater detail in Supplementary Fig. 10.

To investigate whether any genes exhibit unusual patterns of divergence among *P. falciparum*, *P. reichenowi* and *P. gaboni* parasites, we calculated inter-species distances for 4,500 orthologues (Supplementary Data 1). As expected from mtDNA^7^, the pairwise distance between *P. falciparum* and *P. reichenowi* was about fourfold lower than the distance of either species to *P. gaboni*. However, there were four genes for which these relationships were reversed, that is, the *P. falciparum*–*P. reichenowi* distance was about fourfold higher than the *P. falciparum*–*P. gaboni* distance (Fig. 4). These four genes are all located on the same 8 kb segment of chromosome 4 (Fig. 5a) and include two essential invasion genes encoding the reticulocyte-binding-like homologous protein 5 (*RH5*) and the cysteine-rich protective antigen (*CyRPA*)^27^,^28^. To investigate this further, we amplified regions, from both within and outside the 8 kb segment, from additional ape *Laverania* species (Supplementary Table 6, Supplementary Fig. 9). Evolutionary trees derived from the *EBA165* (Fig. 5c) and *GAPM2* (Supplementary Fig. 9c) sequences, which lie immediately beyond the two ends of the 8 kb segment, were consistent with previous topologies^7^. However, trees based on *RH5* (Fig. 5b) and *CyRPA (*Supplementary Fig. 9a) sequences exhibited an unexpectedly close relationship of the *P. falciparum*/*P. praefalciparum* clade with the gorilla parasite *P. adleri*. These discordant relationships indicate a transfer of genetic material from an ancestor of *P. adleri* to an ancestor of *P. praefalciparum*. Mating between members of divergent *Laverania* species is highly unlikely to yield viable offspring (see the ‘Discussion' section), and so the discordant evolutionary history of this 8 kb region is most likely the result of a horizontal transfer of a small genome fragment.

## Discussion

Comparative genomic and population genetic studies require high-quality annotated reference genomes, which have been nearly impossible to obtain for ape *Plasmodium* parasites because of the endangered status of their hosts. Here, we use an SWGA strategy to generate *Plasmodium* genomes from unprocessed blood samples containing large quantities of contaminating host DNA. The selectivity derives from primers that anneal to DNA motifs that are common in the parasite but rare in the host genome, and a polymerase (phi29) that has exceptional processive and strand-displacement functions, which mediate efficient and high-fidelity isothermal DNA amplification^17^,^18^. SWGA obviates the need for mechanical separation of target and background genomes, renders next-generation sequencing more economical and enables the molecular characterization of parasites that cannot be cultured, such as *P. vivax*. Thus, SWGA precludes the need for *in vivo* amplification in non-natural host species, which can result in sequence loss or other artefactual adaptations^29^. SWGA also obviates the need for large blood draws, can be used in resource poor settings, and allows to characterize parasite genomes from dried blood spots. Finally, SWGA can be adapted to other microbes and/or host species, as long as appropriate reference genomes are available for all sample constituents. For example, we have recently amplified *Plasmodium* parasites from infected mosquito DNA^30^, identifying vectors as a potentially valuable source of pathogen genomes. To increase the utility and versatility of SWGA, we are developing an automated pipeline that permits the design, selection and *in silico* evaluation of SWGA primers for different pathogen and host combinations (https://github.com/eclarke/swga).

Although SWGA can generate high-quality *Plasmodium* genomes from samples containing very small quantities of parasite DNA (Supplementary Table 1), the method has limitations. SWGA is not useful for identifying copy number variation because of the uneven distribution of SWGA-derived sequence reads (Fig. 1d). Moreover, uneven coverage precludes the assembly of repetitive genome regions, such as in the subtelomers, since assemblers cannot accurately determine their numbers. SWGA also requires stringent sample preservation. For example, two gorilla samples (SA3066 and SA3157), which were frozen as whole blood without preservation, failed to yield *Laverania* genome sequences on SWGA, although both were low-level parasite DNA positive (0.00073 and 0.00024%, respectively). Nonetheless, when the respective SWGA products were subjected to limiting dilution PCR, gene fragments of several nuclear genes (*GAPM2, CyRPA, FIKK*) were readily amplified (Supplementary Table 6). Thus, SWGA can mediate selective amplification even when samples contain insufficient numbers of parasite genomes required for whole-genome amplification.

The newly generated *P. gaboni* and *P. reichenowi* genomes provided an opportunity to examine levels of genetic polymorphism within ape *Laverania* species, revealing that they were almost 10-fold more diverse than human *P. falciparum*. It has long been suspected that *P. falciparum* has unusually low genetic diversity^31^, although the underlying causes have been the subject of much debate^32^. Genome-wide analysis of human and chimpanzee parasites now show that this low diversity is not a general characteristic of *Laverania* parasites, and therefore not simply an artefact of their very A+T-rich genomes nor a consequence of the recurrent bottlenecks that characterize their life cycle^33^,^34^. The expected neutral nucleotide diversity is dependent on the effective population size, which for a parasite is generally dependent on the population size of its host. Numbers of chimpanzees seem unlikely to have been ten times larger than those of humans in the past^35^, and a simpler explanation for the extremely low diversity in *P. falciparum* is a recent stringent population bottleneck that likely occurred during the cross-species transmission of its gorilla precursor^7^. Importantly, very recent selection for drug resistance cannot explain the genome-wide reduction of *P. falciparum* diversity. Although the spread of resistant alleles has reduced polymorphism in narrow regions immediately surrounding the drug-resistance loci, the high rate of recombination in *P. falciparum* maintains normal diversity levels elsewhere in the genome^36^,^37^.

Previous attempts to date the last common ancestor of *P. falciparum*, that is, to determine a minimum age for the human parasite, have yielded estimates of up to several hundred thousand years ago^38^,^39^,^40^, but all of these made assumptions concerning the *Plasmodium* molecular clock that cannot be substantiated. In contrast, other data, including the time frames of both the opening of niches for the major anthropophilic vectors belonging to the *Anopheles gambiae* species complex^41^ and the spread of *P. falciparum* resistance mutations in Africa^42^, as well as the low probability of maintaining endemic *P. falciparum* infections in human hunter–gatherer populations^43^,^44^, support a much more recent emergence of *P. falciparum*. The time required to generate the observed nucleotide diversity within *P. falciparum* can be estimated from published mutation rates, combined with estimates of the number of replication cycles that the parasites undergo per year. The *P. falciparum* mutation rate has been estimated to be on the order of 1.0–9.7 × 10^−9^ mutations per site per replication cycle^45^,^46^,^47^. Although the time that *P. falciparum* spends in the mosquito as well as the extent of its replication during the human blood stage may vary, various combinations of alternative scenarios suggest a minimum of at least 200 replication cycles per year (P.M.S, unpublished). At fourfold degenerate sites, which are expected to be neutral and thus to reflect mutation rates, we have found an average diversity of 8 × 10^−4^ per site (Table 3). Even considering the lowest estimates of the *P. falciparum* mutation rate and replication numbers, this level of divergence could have readily accumulated within a few thousand years. While this approach is not sufficiently accurate to provide a firm date, it nonetheless indicates that the level of genetic diversity observed among extant *P. falciparum* strains is sufficiently low that it can easily be reconciled with an origin of the human parasites within the past 10,000 years.

To gain insight into the biology of *Laverania* parasites, we examined members of multigene families that function at the host-parasite interface. The previous finding of *var*-like genes in *P. gaboni* indicated that this gene family performs functions essential for all *Laverania* parasites, not only *P. falciparum*^10^. Here we show that the genes encoding members of the FIKK family of protein kinases, which are thought to play a key role in the remodelling of infected erythrocytes, duplicated and diversified rapidly following the emergence of the *Laverania* lineage. Thus, the expansion of the FIKK family from a single gene in non-*Laverania* parasites to up to 22 genes in *Laverania* parasites represents another unique feature of this subgenus (Fig. 3). Interestingly, other exported multigene families that are expanded in *P. falciparum* and *P. reichenowi* also have syntenic orthologues in *P. gaboni* (Supplementary Table 7)^48^. These include the *DNAJ* genes, which encode molecular chaperones in other eukaryotes, but are thought to facilitate interactions with the erythrocyte skeleton and possibly control knob formation in *Laverania* parasites^49^,^50^. These also include genes that encode proteins belonging the poly-helical interspersed subtelomeric subclass b (PHISTb) family, which localize to the erythrocyte periphery^51^ and in some instances interact with proteins of the *P. falciparum* erythrocyte membrane protein 1 (PfEMP1) family that are encoded by *var* genes^24^,^52^. Thus, the rapid expansion and diversification of the *FIKK* gene family likely occurred in concert with other exported multigene families^48^, which may be responsible, at least in part, for the unique biology of *Laverania* parasites, including their ability to mediate RBC cytoadhesion, tissue sequestration and/or host immune escape^8^,^24^.

Although the origin of *P. falciparum* is now well-established, nothing is known about the evolutionary and mechanistic processes that led to its emergence. Genome-wide analysis of the newly derived *P. reichenowi* and *P. gaboni* genomes showed that a short region on chromosome 4, which includes the essential invasion genes *CyRPA* and *RH5*, exhibited anomalous inter-species divergence levels (Figs 4 and 5). Phylogenetic analyses indicate that this reflects an exchange of DNA from an ancestor of one gorilla parasite, *P. adleri*, to the ancestor of another gorilla parasite, *P. praefalciparum*, before the transmission of the latter to humans resulting in *P. falciparum*. While we cannot formally exclude the possibility that the divergent fragment was generated following introgression, several lines of evidence suggest that a horizontal transfer of a small genome fragment is much more likely. First, chimpanzees and gorillas are each infected with three *Laverania* species; since these parasites are sympatric and most wild-living chimpanzees and gorillas are multiply infected^7^, the implication is that there are strong pre- and/or post-reproductive barriers preventing their hybridization. Indeed, if cross-species mating were possible, we would expect to see evidence of this in the genomes of *P. gaboni* and *P. reichenowi*, which is not the case. Second, to go from the initial hybrid containing 50% *P. adleri* DNA, to the current situation with <0.05% *P. adleri* DNA, the introgression process would have required numerous successful generations of backcrossing to *P. praefalciparum*. This seems much less likely than the asexual transfer of a small amount of DNA, especially since cultured erythrocyte-stage parasites of *P. falciparum* are known to take up DNA spontaneously from their host cell cytoplasm^53^ and infected RBCs have been shown to communicate via exosome-like vesicles that are capable of delivering genes^54^. Thus, the horizontal transfer likely occurred during the blood stage infection of a gorilla (or gorilla ancestor) harbouring multiple *Laverania* species^7^.

Recent studies have shown that RH5, CyRPA, the RH5-interacting protein RIPR, and likely other proteins form a multiprotein complex that is attached to the merozoite surface^28^. This adhesion complex ensures the proper positioning of RH5, which lacks a transmembrane domain, thus facilitating its binding to the erythrocyte receptor basigin, an obligate step in the erythrocyte-invasion process^27^. Given the essential nature of these interactions, the acquisition of ‘matching' *RH5*- and *CyRPA*-coding regions on both ends of a mosaic fragment seems unlikely to represent a chance event. Indeed, the initially transferred fragment may have been longer, but could have been reduced in size by successive recombination events, which would have inevitably eaten away at the edges of the region, replacing *P. adleri*-derived sequences with *P. praefalciparum* DNA. This process of erosion would have continued until any further shortening was deleterious because it failed to conserve compatible RH5 and CyRPA proteins. Analyses of the fragment boundaries identified the 5′-break point to lie within the *CyRPA* gene, but very close to the end of the region encoding the predicted signal peptide, which would be expected to be cleaved before binding RH5, providing strong support for this hypothesis (Fig. 5d; Supplementary Fig. 10).

While the adaptive pathways required for the colonization of humans remain to be elucidated, it is tempting to speculate that the horizontal gene transfer of *RH5*, which encodes a major *P. falciparum* host specificity determinant^55^, conferred a fitness advantage that predisposed *P. praefalciparum* to infect humans. However, even if this was the case, this event alone was clearly not sufficient to facilitate the cross-species transmission, since all characterized strains of *P. praefalciparum* carry the same *P. adleri*-derived version of the *RH5* gene. Moreover, the horizontal transfer occurred long before the emergence of *P. falciparum* (Fig. 5b). All available genetic^7^,^56^ and epidemiological^56^,^57^ evidence point to a single gorilla-to-human transmission event, indicating that present-day gorilla parasites do not infect humans. Thus, the *P. praefalciparum* strain that was able to cross the species barrier must have carried one or more highly unusual mutations. It is possible that these mutations included an adaptation to a different mosquito vector, such as the ability to efficiently infect the main human vector *A. gambiae*^58^, when humans transitioned from a hunter–gatherer to a more settled lifestyle. Whole-genome sequencing of *P. praefalciparum*, and functional analyses of gametocyte surface proteins, such as Pfs47 and its *Laverania* orthologues^58^, will be necessary to elucidate this.

## Methods

### Ape samples

Blood samples (5–10 ml) were collected from sanctuary chimpanzees (*Pan troglodytes)* living in outside enclosures in close proximity to wild apes at the Sanaga Yong Chimpanzee Rescue Center in Cameroon (*n*=24) and the Tchimpounga Chimpanzee Rehabilitation Center (*n*=1) in the Republic of the Congo. Members of both the central (*P. t. troglodytes*) and the Nigeria-Cameroonian (*P. t. ellioti*) subspecies were sampled. Blood was obtained for veterinary purposes only or represented leftover specimens from yearly health examinations. None of the chimpanzees exhibited symptoms of malaria at the time of sampling. Most blood samples were preserved in RNAlater (1:1 vol/vol) without further processing, except for 6 samples, which were subjected to density-gradient centrifugation in the field to enrich for RBC (Supplementary Table 6). Briefly, blood was diluted in PBS (1:1 vol/vol), layered over Lymphoprep (Axis-Shield), and then centrifuged at 800 g for 20 min. After removal of the mononuclear cell layer, the purified erythrocytes were preserved in RNAlater (1:1 vol/vol). All samples were transported at ambient temperature and then stored at −80 °C. Small quantities of blood were also obtained from two western gorillas (*Gorilla gorilla*) of unknown geographic origin, who were killed by hunters and confiscated by the anti-poaching programme of the Cameroonian Ministry of Environment and Forestry. Blood was collected from around the inflicted wounds and frozen directly without preservation. Ape faecal samples (*n*=38) were selected from an existing bank of chimpanzee and western gorilla specimens previously shown to contain *Laverania* parasite DNA^7^,^10^,^59^. These specimens were collected from non-habituated apes living in remote forest areas, with a two-letter-code indicating their field site of origin as previously reported^7^,^59^. DNA was extracted from whole blood and RBCs using the QIAmp Blood DNA Mini Kit, the Puregene Core Blood Kit (Qiagen), or the NucliSENS miniMag extraction kit (Biomérieux). Sample collection was approved by the Ministry of Environment and Forestry in Cameroon, and by the Ministry of Forest Economy and Sustainable Development in the Republic of Congo, respectively. All samples were shipped in compliance with Convention on International Trade in Endangered Species of Wild Fauna and Flora regulations and country-specific import and export permits.

### *Laverania* species identification

The *Laverania* species composition of ape blood and faecal samples was determined by limiting dilution PCR (also termed single-genome amplification), followed by phylogenetic analysis, essentially as described^60^. Briefly, DNA was end point diluted such that fewer than 30% of PCR reactions yielded an amplification product (according to a Poisson distribution, a well-yielding PCR product at this dilution will contain only a single-DNA template >83% of the time)^60^. Amplification products were gel purified, and sequenced directly without interim cloning. Sequences containing double peaks, indicative of the presence of multiple templates or early PCR errors, were discarded. In addition to yielding an accurate representation of the *Plasmodium* species present in the sample, this approach also generates sequences devoid of *Taq* polymerase induced misincorporations and template switching. Samples were analysed at mitochondrial, nuclear and apicoplast loci (Supplementary Tables 1 and 6), including portions of cytochrome B (*cytB*), the erythrocyte-binding antigens 165 and 175 (*EBA165*, *EBA175*), the gametocyte surface proteins P47 and P48/45 (*p47*, *p48/45*), the lactate dehydrogenase (*ldh*), the reticulocyte-binding protein homologue 5 (*RH5*), the cysteine-rich protective antigen (*CyRPA*), members of Phe-Ile-Lys-Lys (FIKK) containing protein kinase multigene family (*FIKK7.2, FIKK14* and *FIKK9.15)* and the Clp chaperone PfC10_API0060 (*clpM;* previously termed *clpC*^7^) gene. Primers and PCR conditions have been described^7^,^10^, except for those used for the amplification of *RH5*, *CyRPA* and *FIKK* genes. *RH5* gene fragments (801–869 bp) were amplified using PfrRH5F5 (5′-CRAAGAATCAAGAAAATAATCTGAC-3′) and PfrRH5R5 (5′-GGGACATCATTGAACTTSATTTGTAG-3′) in the first round, and PfrRH5F6 (5′-TTGTTTATKCCTTCTCATAATKCTT-3′) and PfrRH5R6 (5′-CACTTTGTTGTAAAATAYTTGTCATATC-3′) in the second round of PCR. *CyRPA* gene fragments (461–792 bp) were amplified using CyRPA_F1 (5′-TTTYATTTTTTCAAATTGTCTTAGTT-3′) and CyRPA_R1 (5′-ATGTCTCGCCYTTGTCGTG-3′) in the first round, and CyRPA_F2 (5′-GTCRTCATGTTTTYATAAGGACTG-3′) and CyRPA_R2 (5′-CCATACATAAAATGTCATCCTTCTT-3′) in the second round of PCR, or CyRPA5F1 (5′-AAGGACTGARTTRTCGTTYRTAAAG-3′) and CyRPA5R1 (5′-AACKTYCCTCCATARCAACCT-3′) in the first round, and CyRPA5iF2 (5′-TARTGTTCCTTGTRTTSGKGATAT-3′) and CyRPA5iR2 (5′-ATCMCCYACATAAAAATGAAATGAC-3′) in the second round of PCR. The FIKK7.2 fragment (637 bp) was amplified using FIKK7.2_F993 (5′-AAGATTCCTATTARTGCATGGRTAAA-3′) and FIKK7.2_R1782 (5′-ATGATGGATCAGAACGCTTCC-3′) in the first round, and FIKK7.2_F1061 (5′-AAATGCTGAAAATTATGTTATGGAAG-3′) and FIKK7.2_R1724 (5′-GATYCCCAACATATATTTATCAACTG-3′) in the second round of PCR. The FIKK14 fragment (537 bp) was amplified using FIKK14_F1280 (5′-TGAAATGTAGAAGTAGATTAGCAA-3′) and FIKK14_R1965 (5′-GTGTTAAACCTGCTTCATGTAATCTT-3′) in the first round, and FIKK14_F1321 (5′-ACTGTATATAATTGGACRTTAGGTAA-3′) and FIKK14_R1884 (5′-CTAAATCATCATCATCATCATCCATA-3′) in the second round. Finally, the FIKK9.15 fragment (730–733 bp) was amplified using PgSY75FIKK_F1 (5′-CGGATAGAGATGACGTTTCACA-3′) and PgSY75FIKK_R1 (5′-AAGGCACATGCCTCCATAATA-3′) in the first round, and PgSY75FIKK_F2 (5′-ACAGGAGATAATGGAGGAAATGTAG-3′) and PgSY75FIKK_R2 (5′-CCTACCACGTTTACTAAGTCCAATA-3′) in the second round of PCR. For each sample, multiple single-template-derived amplicons were sequenced and their species origin identified by phylogenetic analysis (see GenBank accession codes in Supplementary Table 8). This analysis permitted the identification of samples that represented single (or near single) *Laverania* species infections for SWGA (Supplementary Table 1).

### *Laverania*-specific real-time PCR

To determine the amount of *Laverania* DNA within a blood or faecal sample, DNA was subjected to quantitative (q)PCR using a 7900HT Fast Real-Time PCR System and the Power SYBR Green qPCR kit (Life Technologies). *Laverania*-specific forward (5′-ACATGCCACATGGAAAAGCTT-3′) and reverse (5′-CTGGGGCCTTGGTAAATCCA-3′) primers were used to amplify a 144 bp fragment of the nuclear *ldh* gene. PCR cycling conditions included 2 min at 50 °C, 10 min at 95 °C and 40 cycles of 15 s at 95 °C and 1 min at 60 °C. To estimate the number of genome copies per well, human genomic DNA containing known quantities of purified *P. falciparum* 3D7 DNA was used to generate a standard curve, which was included on all qPCR plates (Supplementary Table 1).

### Design of SWGA primers

In contrast to traditional phi29 whole-genome amplification methods that use random primers to amplify all DNA templates within a sample, SWGA requires primers that bind frequently and evenly across the pathogen genome, but only rarely to the contaminating host DNA. To identify such primers, we determined the frequency of all short sequence motifs (8–12 bp in length) in both a *P. falciparum* (3D7) and human (GRCh37) reference sequence and then calculated the average distance between their locations within these genomes (Supplementary Fig. 1). This approach identified 2,418 motifs that were spaced apart (on average) <50 kb in the *P. falciparum*, but >500 kb in the human genome (Fig. 1a). To select the best possible primers, motifs with a melting temperature (T_m_) below 18 °C and above 30 °C were discarded because they were unlikely to properly anneal to the template DNA. Motifs that contained four or more contiguous self-complementary bases were also eliminated to avoid the formation of homodimers. Finally, motifs predicted to bind greater than three times to human mitochondrial DNA were eliminated, since this circular genome would be disproportionally targeted by phi29 for ‘rolling-circle' amplification. These criteria identified 149 potential SWGA primers.

In a previous study, we found that motifs that exhibited the highest target-to-nontarget binding ratios were able to mediate selective amplification of bacterial genomes from infected host DNA^19^. However, it was unclear whether this criterion alone would be sufficient for more complex (multi-chromosomal) eukaryotic genomes. To design primers capable of amplifying all regions of the *Plasmodium* genome, we developed a metric that scored both selectivity and evenness of coverage. To score a set of primers, we divided the *P. falciparum* and human genomes into 10-kb non-overlapping segments and calculated the proportion of segments that contained at least one primer-binding site (Supplementary Fig. 1). Since our goal was to identify primer-binding sites in as many *P. falciparum* segments as possible, while minimizing segments containing the same binding site in the human genome, we defined our ‘set score' as the difference between the former and the latter (Supplementary Fig. 1).

Starting with a set of 149 primers, there are a total of 1.2 × 10^15^ possible combinations of 10 or fewer primers. Since identifying the single best set would be computationally impossible, we used a heuristic approach to search for optimal primer combinations. Reasoning that heterodimer formation would reduce amplification efficiency, we divided the 149 primers into eight mutually exclusive groups, where no two primers contained four or more contiguous complementary bases. For each group, we first scored primers individually and selected the highest scoring primer. We then paired this primer with all other primers and identified the highest scoring pair. This process was repeated by iteratively adding primers until the set score no longer improved (Supplementary Fig. 1). Applying this approach to all primer groups generated eight high-scoring sets. The two best sets, including primer set 6A (5′-TAAATAAAAA*A*A-3′, 5′-CATAAAAAA*A*A-3′, 5′-TAAATAATAA*T*A-3′, 5′-ATCATAATA*A*T-3′, 5′-TAACAAAAAA*A*A-3′, 5′-TAATAAATAA*A*A-3′, 5′-TAACATAGG*T*C-3′, 5′-TAGTAGTAG*T*A-3′, 5′-ATAATAAATA*A*T-3′, 5′-CATAATAATA*A*T-3′) and primer set 8A (5′-TTTTTTTATT*T*A-3′, 5′-TATTATTATT*T*A-3′, 5′-TTTTTTTAT*G*T-3′, 5′-ATTATTATG*A*T-3′, 5′-TTTTTTTTGT*T*A-3′, 5′-TATTTATTAT*T*A-3′, 5′-GACCTATG*T*TA-3′, 5′-TACTACTAC*T*A-3′, 5′-TATTATTTAT*T*A-3′, 5′-TATTATTATT*G*T-3′) were then tested using human genomic DNA samples spiked with known quantities of *P. falciparum* DNA (Fig. 1c). All primers contained phosphorothioate bonds between the two most 3′ nucleotides (indicated by asterisks) to prevent primer degradation by phi29. A primer design pipeline that is applicable to the genomes of other organisms is under development (https://github.com/eclarke/swga).

### SWGA protocol and validation of primer sets

SWGA was performed essentially as described^19^, following established phi29 amplification protocols, but using primers designed to selectively amplify *Laverania* genomes (Fig. 1). Amplification conditions included a 1 h ramp-down step (35 °C to 30 °C), followed by a 16 h amplification step at 30 °C. Phi29 was then denatured for 10 min at 65 °C, and the SWGA product was stored at 4 °C. To validate the SWGA primers, genomic DNA extracted from cultured *P. falciparum* parasites (a subclone of NF54 generated by Kirk Deitsch, Weill Cornell Medical College, which is isogenic with 3D7) and human CD4 T cells (obtained from the Human Immunology Core of the University of Pennsylvania Center for AIDS Research) were mixed to generate human DNA preparations containing 5, 1, 0.1, 0.01 and 0.001% *P. falciparum* DNA. SWGA was performed in a volume of 50 μl using 50 ng of DNA, 3.5 mM of each SWGA primer (set 6A), 1 × phi29 buffer (New England Biolabs), 1 mM dNTPs and 30 units of phi29 polymerase (New England Biolabs). In all, 4 μl of the resulting SWGA product was then subjected to a second round of SWGA using the same amplification conditions, but a different set of primers (set 8A). Each of the human/*P. falciparum* DNA mixtures was amplified separately and purified using Agencourt AmpureXP beads (Beckman Coulter). In all, 20 ng of the resulting SWGA products were used to generate short-insert libraries (Nextera Library Prep Kit) and sequenced on an Illumina MiSeq, yielding 150 bp paired reads. Enrichment was quantified by mapping paired reads first to the human and then to the *P. falciparum* 3D7 genome using SMALT 0.7.6 and then calculating the percentage of reads that mapped to *P. falciparum* 3D7 (https://www.sanger.ac.uk/resources/software/smalt/).

To determine the efficiency of SWGA, we performed a rarefaction analysis, examining both the selectivity and evenness of amplification for different ratios of host/parasite DNA. For each human/Pf DNA mixture, subsets of reads were randomly selected and mapped to the *P. falciparum* (3D7) and human reference genomes (GRCh37) simultaneously. The per cent of the *P. falciparum* genome with ≥1 × coverage was then calculated and compared with the expected coverage of the same unamplified human/Pf mixture (Fig. 1c).

### Selective amplification of *P. reichenowi* and *P. gaboni* genomes

To amplify near-full-length *Laverania* parasite genomes from unprocessed ape blood, we selected one chimpanzee sample (SY57) that contained mostly (>99%) *P. reichenowi* and two others (SY75 and SY37) that contained exclusively *P. gaboni* DNA for SWGA analysis (Supplementary Table 1). Since these samples contained very little *Laverania* DNA (0.00081–0.14%), we first digested them with methylation-dependent restriction enzymes (MspJI and FspEI) to selectively cleave the contaminating host DNA^20^. Briefly, 200 ng to 1 μg of total DNA were digested with FspEI (5 U) and MspJI (5 U) for 7 h at 37 °C, after which the enzymes were heat inactivated. The digestion products were purified and subjected to two successive rounds of SWGA using the same conditions as described above. For each chimpanzee sample, SWGA was performed using multiple DNA replicates (Supplementary Table 1), with half being first amplified with primer set 8A followed by primer set 6A, and the other half being first amplified with primer set 6A followed by primer set 8A. Amplification products were purified, pooled and used to generate short-insert libraries (650 bp) using the Illumina TruSeq PCR-Free Library Preparation Kit (Supplementary Tables 1 and 9). To facilitate subsequent genome assembly, we also generated long-insert libraries (3 kb, 5 kb, 8 kb and 9 kb) for the *P. gaboni* sample SY75 (Illumina Nextera Mate Pair Sample Preparation Kit). All libraries were sequenced using the Illumina MiSeq and paired reads were first mapped to the chimpanzee reference genome (Pan_troglodytes-2.1.4) using SMALT. The remaining reads were then mapped to the *Plasmodium* genome, with Pf3D7 serving as the reference for SY75 and SY37, and PrCDC serving as the reference for SY57. Although SY75 (73%) and SY37 (61%) yielded fewer parasite**-specific reads than SY57 (89%), this was not due to a reduction in amplification selectivity, but reflected the difficulty of mapping *P. gaboni* reads to the much more divergent *P. falciparum* genome (Supplementary Table 1). Illumina sequencing runs and accession codes are listed in Supplementary Table 9.

### Assembly of *P. gaboni* and *P. reichenowi* draft genomes

Draft genomes were generated for the *P. reichenowi* strain PrSY57 and the *P. gaboni* strain PgSY75 using reference-guided *de novo* assembly with post-assembly genome improvements^12^,^21^. First, working drafts of the PrSY57 and PgSY75 genomes were generated by iteratively mapping (non-chimpanzee) reads to the PrCDC and Pf3D7 references, respectively, using Geneious 6 (Biomatters Limited, http://www.geneious.com). This mapping process, which was repeated 10 times, resulted in a sequence that represented the read mapping consensus at all positions with greater than or equal to fivefold coverage. At positions with lower coverage, the sequence of the reference (Pf3D7 or PrCDC) was used instead. All reads were then re-mapped to this consensus using two iterations. The resulting draft reference represented the mapping consensus at all positions with greater than or equal to fivefold coverage, with positions with less than fivefold coverage denoted by ‘N's.

Before *de novo* assembly, error correction was performed on short-insert libraries from each sample using String Graph Assembler (SGA 0.10.12, https://github.com/jts/sga) as previously described^12^. For the *P. gaboni* sample PgSY75, reads were also normalized using KHMER^61^, which uses k-mer frequencies to estimate and normalize genome coverage in a reference-free manner, thus facilitating subsequent *de novo* assembly. This process yielded 11 million reads.

After mapping reads to the working draft reference using SMALT (http://sourceforge.net/projects/smalt/), a reference-guided *de novo* assembly was generated using the Columbus extension to Velvet 1.1.06 (http://www.ebi.ac.uk/~zerbino/velvet/). Assemblies were produced using a variety of k-mer lengths and coverage settings. Comparing these assemblies with the Pf3D7 and PrCDC references, we identified several tandem duplications, which on visual inspection were judged to likely represent assembly errors. We thus changed the assembly parameters to minimize the number of these duplications. Specifically, we varied k-mer length, coverage cutoff and minimum paired coverage, and analysed the resulting assembly quality by comparing the length of contigs, maximum node length, total assembly length and the number of tandem duplications compared with the reference genome.

For the *P. gaboni* sample PgSY75, contigs produced by Velvet Columbus were further scaffolded using long-insert libraries with SSPACE 2.0 (http://www.baseclear.com). Scaffolding was performed iteratively, first using the 3-kb library, then the 5-kb library and finally the 8- and 9-kb libraries. Scaffolding was performed using default parameters, except for (i) a minimum number of mate pairs (-k) of 10 for the 3-kb library and 5 for the 5-, 8- and 9-kb libraries, respectively, (ii) a maximum ratio between the two best pairs (-a) of 0.6, (iii) a minimum required overlap (-n) of 60 bp and (iv) a minimum contig size (-z) of 500. Scaffolding was not performed for the *P. reichenowi* PrSY57 because long-insert libraries were not generated for this sample.

To improve the quality of the draft references, contigs and scaffolds produced by Velvet Columbus and SSPACE were subjected to two iterations of post-assembly improvement using PAGIT v1 (ref. 21). Contigs were aligned against the respective reference genomes using ABACAS 1.3.1 (http://abacas.sourceforge.net) and joined into a single-ordered sequence separated by gaps (‘N's). The resulting ordering was compared with the reference genome using blastn to identify erroneously placed contigs. ABACAS parameters for minimum per cent identity (-i) and minimum contig coverage (-v) were varied to maximize the total number of correctly placed contigs (for example, -i 90 was used to minimize *P. gaboni* contamination in the *P. reichenowi* SY57 assembly). Contigs were then manually rearranged in the Artemis Comparison Tool^62^ to correct any remaining placement errors. Gaps between contigs were closed using gapfiller 1.10 (ref. 63) and IMAGE 2.4.1 (http://sourceforge.net/projects/image2/). Since the closing of gaps also produced tandem duplications, parameters for gapfiller and IMAGE were varied to minimize the number of duplications and maximize the number of gaps closed.

Mapping paired reads to the improved draft genome identified several instances where Velvet or gap-closure produced erroneously assembled sequence. Since read coverage is often reduced on both sides of an assembly error, we calculated the mean read coverage for a 1,750 bp window surrounding these positions (the central 750 bp which were slightly larger than the library insert size were excluded from these calculations). We then broke the draft genome into contigs at positions where the coverage was either below five paired reads or 10% of the mean coverage of the 1,750 bp window, and repeated the process of contig ordering and gap-closure using the broken contigs, varying the same parameters as before.

The ordered, gap-closed, draft genome produced by PAGIT was corrected using iCORN2 (http://icorn.sourceforge.net), which corrects SNP and indel errors based on the read consensus. We ran iCORN iteratively until no additional corrections of the genome were required. The final output was designated version 0.1 of both the PgSY75 and PrSY57 draft chromosomal assemblies, with all additional edits made after manual inspection during gene annotation and subsequent analyses.

### Generation of PgSY75 and PrSY57 unplaced read bins

All contigs that could not be placed into chromosomal scaffolds of an assembly during the PAGIT process were put into an unplaced-read-bin (version 0.1). These ‘bins' were then expanded using *de novo* assemblies of (non-chimpanzee) reads that failed to map to both the chromosomal assembly and the v0.1 bin. This was done by mapping all reads from PgSY75 and PrSY57 to their respective draft assembly and bin using SMALT, and then performing *de novo* assembly of the remaining unplaced read pairs using SPAdes 3.1.1 (ref. 64). SPAdes was run using the default multicellular mode parameters, except for the k-mer length (-k) that was set to 21, 33, 55 and 77. For PgSY75, the resulting contigs were corrected using iCORN, using only unmapped read pairs from the previous step and added to the unplaced bin. For PrSY57, the combined unplaced bin contigs were screened for contaminating *P. gaboni* sequences by performing blastn searches to a combined database of PrCDC, Pf3D7 and PgSY75 chromosomes. Contigs were only retained if their best match was to a *P. reichenowi* contig, exhibited ≥90% identity, and had an E-value ≤10^−15^. Duplicated contigs, which had been assembled erroneously due to the presence of inter-strain polymorphisms or sequencing error, were initially merged by running dipSPAdes^65^ on the combined unplaced contig bins for each draft assembly, using haplocontig mode. Each unplaced contig in the reduced bin was then compared with the chromosomal assembly and other unplaced bin contigs using blastn, and those that were >85% identical to chromosomal or bin contigs were aligned to their match, visually inspected and either removed or used to improve the existing assembly. The resulting de-duplicated bin was combined with the v0.1 draft chromosomal assembly and designated the v0.1 draft genome.

### Annotation of the PgSY75 and PrSY57 draft genomes

Annotations were transferred to the PgSY75 and PrSY57 draft genomes from *P. falciparum* (Pf3D7) and *P. reichenowi* (PrCDC) reference genomes, respectively. Annotation transfer was performed using RATT (http://ratt.sourceforge.net) and corrected manually in the Artemis Comparison Tool^62^ using a blastn alignment to the corresponding reference. Genes in the draft genomes that were not present in Pf3D7 or PrCDC, or had been missed by RATT, were identified by *de novo* annotation in Augustus^66^ using the *P. falciparum* species configuration. *De novo* annotations that overlapped transferred annotations were removed. The remaining *de novo* annotations were compared with their reference strains using blastn and tblastx to identify putative orthologues and homologues, and corrected by visual inspection. Annotations for which no homologue could be identified in the reference were compared individually with all available *Plasmodium* genomes, and deleted if no putative homologue could be found.

### Generation and annotation of the PgSY37 draft genome

Because the small amounts of *P. gaboni* DNA present in sample SY37 resulted in greater unevenness of whole-genome amplification and sequence coverage, the PgSY37 draft genome was assembled by iteratively mapping the SWGA generated sequencing reads to the PgSY75 genome, using the same methods and parameters described above. Unplaced reads were assembled using SPAdes^64^ and placed into the PgSY37 unplaced read bin. The PgSY37 genome was annotated by strain level annotation transfer from the PgSY75 genome using RATT, and corrected by visual inspection.

### Genes used in genome-wide analyses

Syntenic orthologues in *P. falciparum* 3D7 and *P. reichenowi* CDC were identified by chromosomal alignment. After exclusion of (i) *var*, *rif* and *stevor* gene families, (ii) genes that were pseudogenes in at least one of these *Laverania* species (Supplementary Table 3), (iii) genes that had previously been suggested to be dimorphic in *P. falciparum* (*msp1*, *msp2*, *msp3*, *msp6* and *EBA175*), and (iv) genes for which orthologues could not be identified in *P. gaboni* (Supplementary Data 1), the remaining sets of orthologues were used for genome-wide analyses. Subtelomeric regions, which were excluded from *P. falciparum* polymorphism data, were defined as regions at the ends of chromosomes that consisted primarily of genes previously annotated as subtelomeric or members of subtelomeric gene families, including *var*, *rif*, *stevor*, *PHIST*, *mc-2tm, hyp* gene families 1–17, *resa*, lysophospholipase, *DNAJ* and acyl-coA synthetase. Subtelomeric genes are identified in Supplementary Data 1.

### Inter-species divergence

The lengths of coding sequences from the annotated genomes were compared with their homologues or orthologues in the respective reference sequence (PrCDC for PrSY57, Pf3D7 for PgSY75 and PgSY37). Genes were only included in genome-wide analyses if they (i) were ≥90% of the length of the reference homologue/orthologue or (ii) were ≥80% of the length of the reference orthologue/homologue, but also lacked assembly gaps. Each coding region was translated and queried for amino acid repeats using tblastx. Repeated sequences were masked if they comprised at least 20 amino acids with at least 95% identity between repeat units. Low-complexity amino acid sequences were identified in translations using segmasker (NCBI BLAST+package) using default settings, and masked in the corresponding nucleotide sequences. Masked nucleotide sequences were aligned using TranslatorX and MUSCLE. After alignment, any position that was masked, or contained an assembly or alignment gap, was masked in all sequences. Pairwise inter-species genetic distances were calculated in R using the ape package^67^ with the TN93 model of DNA evolution. Genes with unusually high or low inter-species distances were manually inspected and the respective alignments or masked regions were corrected if necessary. If the best alignment required insertion of a gap not divisible by three, the gene was excluded from intra-species diversity analyses (since these required sequence translations). Inter-species distances were calculated using all available orthologues for Pf3D7, PrCDC, PrSY57, PgSY75 and PgSY37.

### Intra-species diversity

For *P. falciparum*, intra-species diversity was calculated using previously published parasite sequence data sets of geographically diverse field isolates collected in Bangladesh, Cambodia, DRC, Gambia, Ghana, Guinea, Laos, Myanmar, Nigeria, Thailand, Kenya and Vietnam (Pf3k 1.0 pilot data release, http://www.malariagen.net/data/pf3k-1). For each country, three samples were chosen at random, reads were mapped to the 3D7 reference, and SNP variant calls were generated for all *P. falciparum* strains simultaneously using the GATK 3.1-1 UnifiedGenotyper after indel realignment. To differentiate true variants from sequencing or alignment artefacts, 354 variant calls were randomly selected and true variants identified by visual inspection. The GATK values (QUAL, QD, ReadPosRankSum, Genotype Quality, FS, BaseQRankSum, MQRankSum) were then compared for each true and artefactual variant, and appropriate cutoffs were selected to minimize false variant calls. Using only SNPs from the core genome, the number of *P. falciparum* strains present in each sample was estimated using estMOI^68^, with one likely mono-infection selected for each country (ERS174561, Bangladesh; ERS050887, Cambodia; ERS347597, DRC; ERS010044, Gambia; ERS157479, Ghana; ERS042044, Guinea; ERS174601, Laos, ERS143480, Myanmar; ERS199640, Nigeria; ERS224908, Thailand; ERS143467, Vietnam). No Kenyan strain was selected since all available samples were likely to represent multi-strain infections. After exclusion of subtelomeric genes, alleles from polymorphic sites were extracted from variant call format (.vcf) files; sites at which three or more samples had missing data (that is, no genotype called) or where the majority genotype was represented by <80% of mapped reads, were excluded from the analysis; otherwise samples with missing data were assumed to have the reference allele. Intra-species diversity (*π*) was determined by calculating the mean number of differences per site for all pairwise combinations of 11 *P. falciparum* strains plus the 3D7 reference. Sites masked in 3D7 (see above) were excluded from intra-species diversity calculations. For *P. gaboni* and *P. reichenowi*, intra-species diversity was calculated from the alignments used for inter-species genetic distance calculations, using the ape R package to count the proportion of non-masked sites that differed between the two strains available for each species (PrCDC and PrSY57 for *P. reichenowi*, PgSY75 and PgSY37 for *P. gaboni*).

### Phylogenetic analyses

Nucleotide sequences used for phylogenetic analyses were aligned using CLUSTAL W, followed by manual correction when necessary. Regions that could not be unambiguously aligned were removed from further analyses. Maximum likelihood phylogenetic analyses were conducted using PhyML^69^, with iterative model fitting based on a class of evolutionary models selected using Modeltest^70^. For the analyses of the *FIKK* orthologues, pseudogene nucleotide sequences were translated, with indels corrected and in-frame stops coded as ‘X', and the deduced amino acid sequences were aligned using MUSCLE. On the basis of this alignment, the conserved FIKK protein regions were identified. The corresponding nucleotide sequences were then codon aligned, guided by the amino acid alignment. To eliminate possible mutational saturation at third codon position sites, these were removed before phylogenetic analyses using PhyML^69^.

## Additional information

**Accession codes:** The whole-genome sequences of PgSY75 and PrSY57 have been deposited in the GenBank BioProject database under the project accession number PRJNA295394. All short-read data have been deposited in the Short Read Archive (SRA) under the accession numbers SRR2414471 to SRR2414493. Limiting dilution PCR-derived sequences have been deposited in GenBank Nucleotide database under the accession codes KT824252 to KT824280, KT824282, KT824286, KT824288 to KT824294, KT824297 to KT824304, KT824306 to KT824312, KT824316, KT824319, KT824322, KT824323, KT824325 to KT824334, KT824337, KT824338, KT824341, KT824342, KT824344, KT824349, KT824350, KT824352, KT824355 to KT824360, KT824365, KT824371, KT824373 to KT824425, KU193795 to KU193804 and KU302812.

**How to cite this article:** Sundararaman, S. A. *et al*. Genomes of cryptic chimpanzee *Plasmodium* species reveal key evolutionary events leading to human malaria. *Nat. Commun.* 7:11078 doi: 10.1038/ncomms11078 (2016).

## Supplementary Material

Supplementary Information Supplementary Figures 1-10, Supplementary Tables 1-9 and Supplementary References

## Figures and Tables

**Table 1:** Selective whole-genome amplification of *P. falciparum* from mixtures of human and parasite DNA.

Per cent parasite admixture*	Total DNA (ng)	*P. falciparum* DNA (ng)	*P. falciparum* genome copies	Total MiSeq reads	Reads mapping to the human genome	Per cent human reads	Reads mapping to the *P. falciparum* genome	Per cent *P. falciparum* reads	Unmapped reads	Per cent unmapped reads	Fold parasite enrichment
5 *P. falciparum*	50	2.5	96,507	3,157,170	9,156	0.3	2,968,254	94.0	179,760	5.7	19
				2,570,124	7,064	0.3	2,408,319	93.7	154,741	6.0	19
1 *P. falciparum*	50	0.5	19,301	3,412,530	34,936	1.0	3,152,623	92.4	224,971	6.6	92
				2,804,890	22,190	0.8	2,660,365	94.8	122,335	4.4	95
0.1 *P. falciparum*	50	0.05	1,930	3,422,726	43,108	1.3	3,174,382	92.7	205,236	6.0	930
				2,638,548	56,552	2.1	2,444,992	92.7	137,004	5.2	930
0.01 *P. falciparum*	50	0.005	193	3,917,388	332,468	8.5	3,390,560	86.6	194,360	5.0	8,700
				3,362,418	429,008	12.8	2,730,631	81.2	202,779	6.0	8,100
0.001 *P. falciparum*	50	0.0005	19	2,430,994	590,934	24.3	1,688,947	69.5	151,113	6.2	69,000
				2,635,560	613,656	23.3	1,832,657	69.5	189,247	7.2	70,000

^*^For each parasite admixture, results are shown for two technical replicates.

**Table 2:** Genome features of *P. gaboni* and *P. reichenowi*.

	*P. reichenowi*	*P. gaboni*	*P. gaboni*
Genome ID	PrSY57	PgSY75	PgSY37
Chromosomal assembly (bp)*	18,852,800	18,463,354	15,330,638
Chromosomal contigs	1,012	331	NA†
Unplaced assembly (bp)‡	798,479	1,474,057	8,902,276
Unplaced contigs	762	818	14,793
Chromosomes	14	14	14
GC content (%)	18.6	18.3	17.1
			
Core protein-coding genes§	4,670 (98.3%)	4,689 (98.7%)	4,071 (85.7%)
Full-length||	4,359 (91.8%)	4,381 (92.2%)	3,295 (69.4%)
Partial¶	311 (6.5%)	308 (6.5%)	776 (16.3%)
			
Subtelomeric protein-coding genes§	235 (23.8%)	222 (33.2%)	108 (16.2%)
Full-length||	182 (18.5%)	189 (28.3%)	72 (10.8%)
Partial¶	53 (5.4%)	33 (4.9%)	36 (5.4%)
			
Other protein-coding genes#	15	51	0
Full-length||	14	44	NA
Partial¶	1	7	NA
tRNA genes	42 (93.3%)	43 (95.6%)	32 (71.1%)
			
rRNA genes	8 (47.1%)	11 (43.3%)	2 (7.7%)
Full-length||	4 (23.5%)	10 (38.5%)	2 (7.7%)
Partial¶	4 (23.5%)	1 (3.8%)	0
			
ncRNA genes	71 (75.5%)	67 (65.7%)	49 (48.0%)
Full-length||	66 (70.2%)	61 (59.8%)	40 (39.2%)
Partial¶	5 (5.3%)	6 (5.9%)	9 (8.8%)
			
Apicoplast genes	45 (76.3%)	58 (85.3%)	NA
Full-length||	27 (90.0%)	30 (100%)	NA
Partial¶	2 (6.7%)	0	NA
tRNA genes	16 (59.3%)	26 (76.5%)	NA
rRNA genes	0	2 (50%)	NA

bp, base pair; NA, not available.

^*^Length of all contigs that could be placed in chromosomes, excluding gaps.

^†^The PgSY37 genome was generated by iteratively replacing the PgSY75 genome with PgSY37 reads and replacing the regions that lacked fivefold coverage with Ns; reads not mapped to PgSY75 chromosomes were assembled *de novo* to generate ‘unplaced contigs'.

^‡^Length of all contigs that could not be placed in chromosomes (bin), excluding gaps.

^§^Gene counts excluding splice variants, but including pseudogenes and partial genes; parentheses indicate the percentage of genes covered in the *Plasmodium* references Pf3D7 (PgSY75 and PgSY37) and PrCDC1 (PrSY57).

^||^Number includes all genes that comprise ≥90% of the lengths of their Pf3D7 or PrCDC orthologues/homologues, as well as all genes that comprise ≥80% of the lengths of their Pf3D7 or PrCDC orthologues/homologues and contain no assembly gaps.

^¶^All annotated coding sequences for which homologues could be identified by BLAST search, but did not contain a sufficiently long sequence to be considered full-length.

^#^Genes for which an orthologue could not be unambiguously identified in the reference genome.

**Table 3:** Nucleotide diversity within *Laverania* species.

Species*	*N*†	*π*‡	*π*4§	Genes	*π*‡	*π*4§	Genes
*P. falciparum*	12	0.00049	0.00081	4,734	0.00043	0.00079	3,111
*P. reichenowi*	2	0.00364	0.00899	4,439	0.00324	0.00876	3,111
*P. gaboni*	2	0.00406	0.01069	3,331	0.00381	0.01049	3,111

^*^Values represent mean values across genes, weighted by the number of sites in the gene. Values at the right are for 3,111 genes available for all three species, with the same set of sites used for each.

^†^Number of strains.

^‡^Pairwise nucleotide diversity across all non-masked coding sites.

^§^Pairwise nucleotide diversity across non-masked fourfold degenerate sites.
