# Supplementary material for: Genomes of cryptic chimpanzee Plasmodium species reveal key evolutionary events leading to human malaria
Source: Nat Commun. 2016 Mar 22;7:11078. doi: 10.1038/ncomms11078 (PMC4804174; doi:10.1038/ncomms11078)
Supplement: Supplementary Information — Supplementary Figures 1-10, Supplementary Tables 1-9 and Supplementary References [file ncomms11078-s1.pdf]

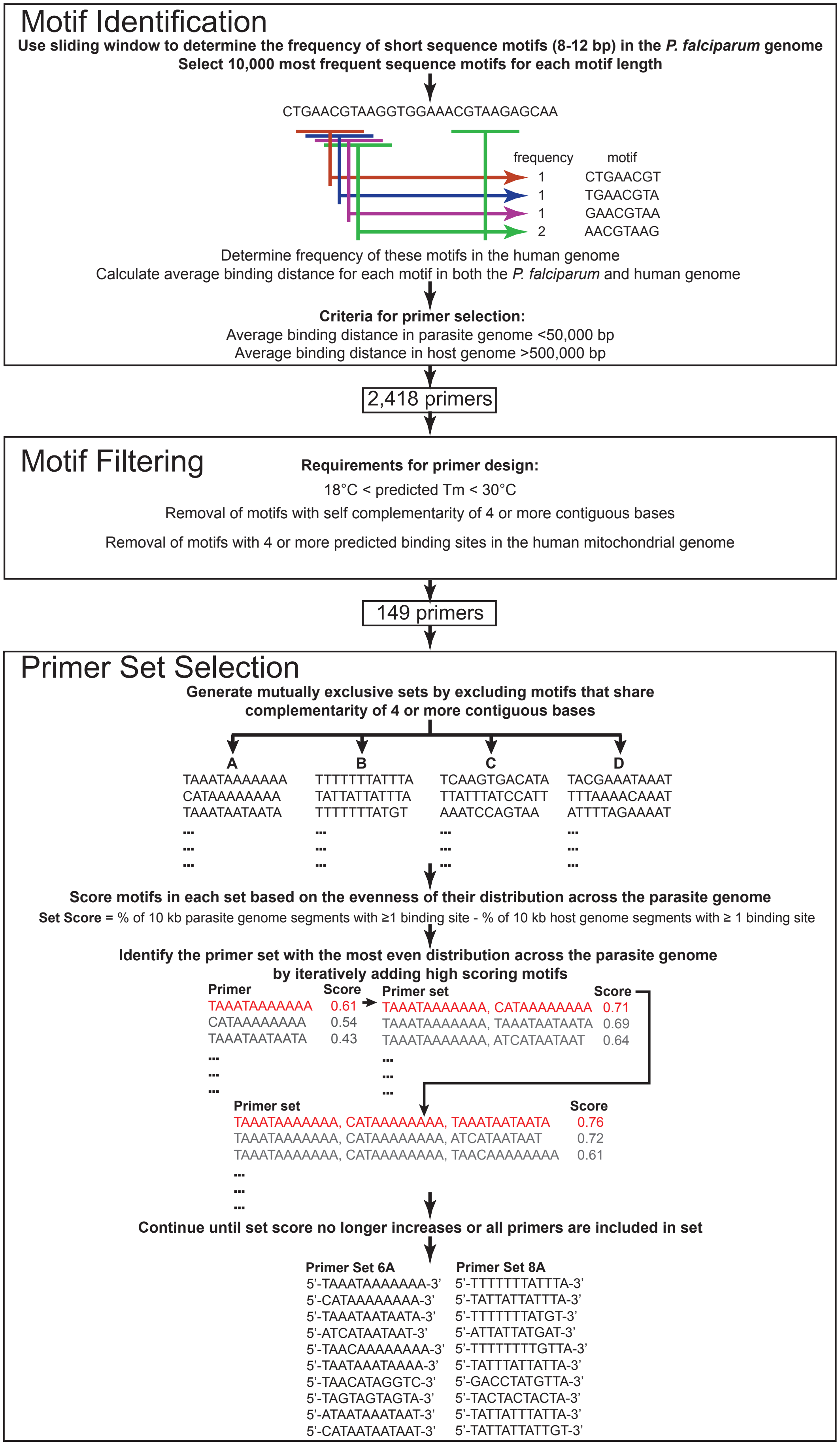
Motif Identification
Use sliding window to determine the frequency of short sequence motifs (8-12 bp) in the P. falciparum genome
Select 10,000 most frequent sequence motifs for each motif length
CTGAACGTAAGGTGGAAACGTAAGAGCAA
frequency
motif
1 CTGAACGT
1 TGAACGTA
1 GAACGTAA
2 AACGTAAG
Determine frequency of these motifs in the human genome
Calculate average binding distance for each motif in both the P. falciparum and human genome
Criteria for primer selection:
Average binding distance in parasite genome <50,000 bp
Average binding distance in host genome >500,000 bp
2,418 primers
Motif Filtering
Requirements for primer design:
18°C < predicted Tm < 30°C
Removal of motifs with self complementarity of 4 or more contiguous bases
Removal of motifs with 4 or more predicted binding sites in the human mitochondrial genome
149 primers
Primer Set Selection
Generate mutually exclusive sets by excluding motifs that share complementarity of 4 or more contiguous bases
A
TAAATAAAAAAA
CATAAAAAAAAA
TAAATAATAATA
B
TTTTTTTATTTA
TATTATTATTTA
TTTTTTTATGT
C
TCAAGTGACATA
TTATTTATCCATT
AAATCCAGTAA
D
TACGAAATAAAT
TTTAAAACAAAT
ATTTTAGAAAAT
Score motifs in each set based on the evenness of their distribution across the parasite genome
Set Score = % of 10 kb parasite genome segments with ≥1 binding site - % of 10 kb host genome segments with ≥ 1 binding site
Identify the primer set with the most even distribution across the parasite genome by iteratively adding high scoring motifs
Primer
Score
TAAATAAAAAAA 0.61
CATAAAAAAAAA 0.54
TAAATAATAATA 0.43
Primer set
Score
TAAATAAAAAAA, CATAAAAAAAAA 0.71
TAAATAAAAAAA, TAAATAATAATA 0.69
TAAATAAAAAAA, ATCATAATAAT 0.64
Primer set
Score
TAAATAAAAAAA, CATAAAAAAAAA, TAAATAATAATA 0.76
TAAATAAAAAAA, CATAAAAAAAAA, ATCATAATAAT 0.72
TAAATAAAAAAA, CATAAAAAAAAA, TAACAAAAAAAA 0.61
Continue until set score no longer increases or all primers are included in set
Primer Set 6A
5’-TAAATAAAAAAA-3’
5’-CATAAAAAAAAA-3’
5’-TAAATAATAATA-3’
5’-ATCATAATAAT-3’
5’-TAACAAAAAAAA-3’
5’-TAATAAATAAAA-3’
5’-TAACATAGGTC-3’
5’-TAGTAGTAGTA-3’
5’-ATAATAAATAAT-3’
5’-CATAATAATAAT-3’
Primer Set 8A
5’-TTTTTTTATTTA-3’
5’-TATTATTATTTA-3’
5’-TTTTTTTATGT-3’
5’-ATTATTATGAT-3’
5’-TTTTTTTTGTTA-3’
5’-TATTTATTATTA-3’
5’-GACCTATGTTA-3’
5’-TACTACTACTA-3’
5’-TATTATTTATTA-3’
5’-TATTATTATTGT-3’

Supplementary Figure 1 Design of SWGA primers for *Laverania* parasites. A sliding window was used to determine the frequency of all possible sequence motifs, 8 to 12 bp in length, within the *P. falciparum* (Pf3D7) genome. The 10,000 most frequent motifs (for each length) were then selected and their average binding distances calculated for both Pf3D7 and a human reference genome (GRCh37). This analysis identified 2,418 motifs that were spaced (on average) less then 50,000 bp apart in the *P. falciparum* genome, but more than 500,000 bp apart in human genome (Fig. 1a). These were further down-selected based on their predicted melting temperature (Tm), degree of self-complementarity, and binding to the human mitochondrial genome. The remaining 149 motifs were subdivided into mutually exclusive groups that lacked inter-motif complementarity of 4 or more nucleotides, and were scored based on their evenness of distribution across the *P. falciparum* genome (calculated as the proportion of 10 kb *P. falciparum* segments containing one or more binding sites minus the proportion of 10 kb human genome segments containing one or more of these same binding sites). Motifs were then combined into sets by iteratively adding motifs and scoring the combination for evenness of distribution across the *P. falciparum* genome. This process was repeated for each set until the score no longer improved. The two highest scoring sets (6A and 8A), each containing 10 primers, were selected for experimental validation using human DNA spiked with known quantities of *P. falciparum* DNA (Fig. 1c).

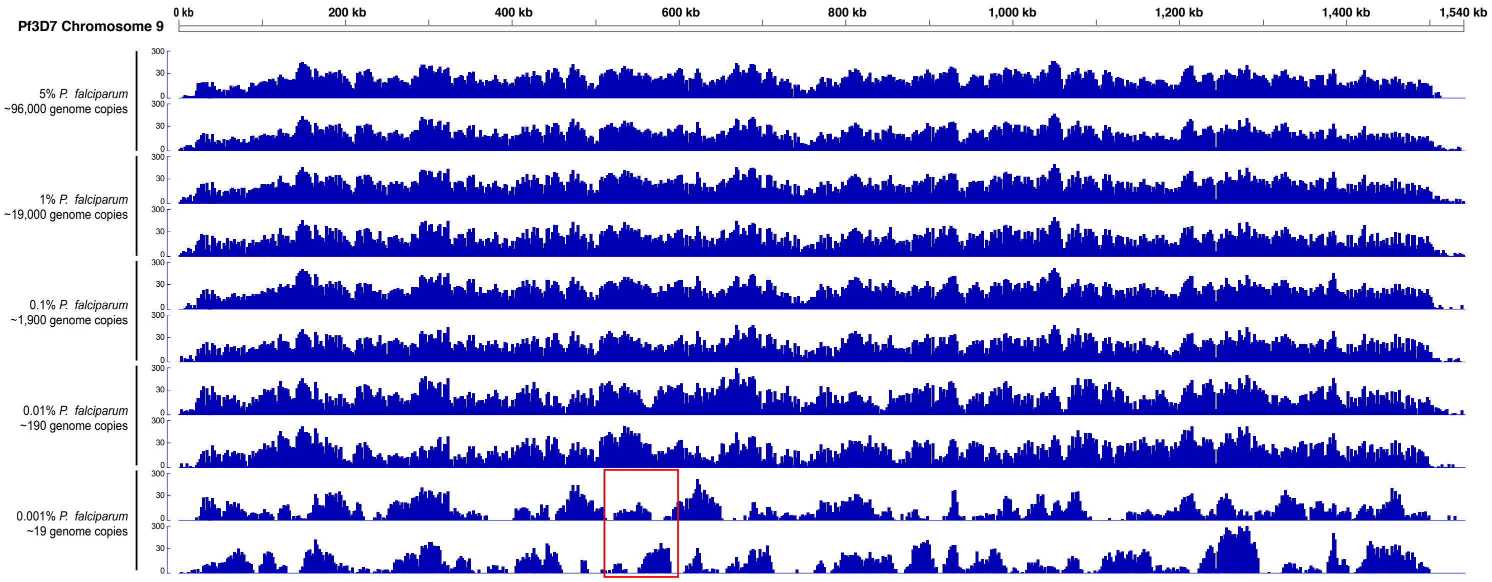

Supplementary Figure 2 Sequence coverage of *P. falciparum* after selective whole genome amplification of spiked human DNA. The MiSeq read depth is shown across chromosome 9 of the Pf3D7 genome (drawn to scale as indicated on top). For each parasite/human DNA mixture, the percentage of *P. falciparum* and estimated number of genome copies are indicated (results from two technical replicates are shown). The red box highlights one example of uneven amplification of a ~50 kb region in two independent SWGA replicates at the lowest *P. falciparum* concentration (19 genome equivalents). The MiSeq read depth across the entire Pf3D7 genome is shown in Fig. 1d.

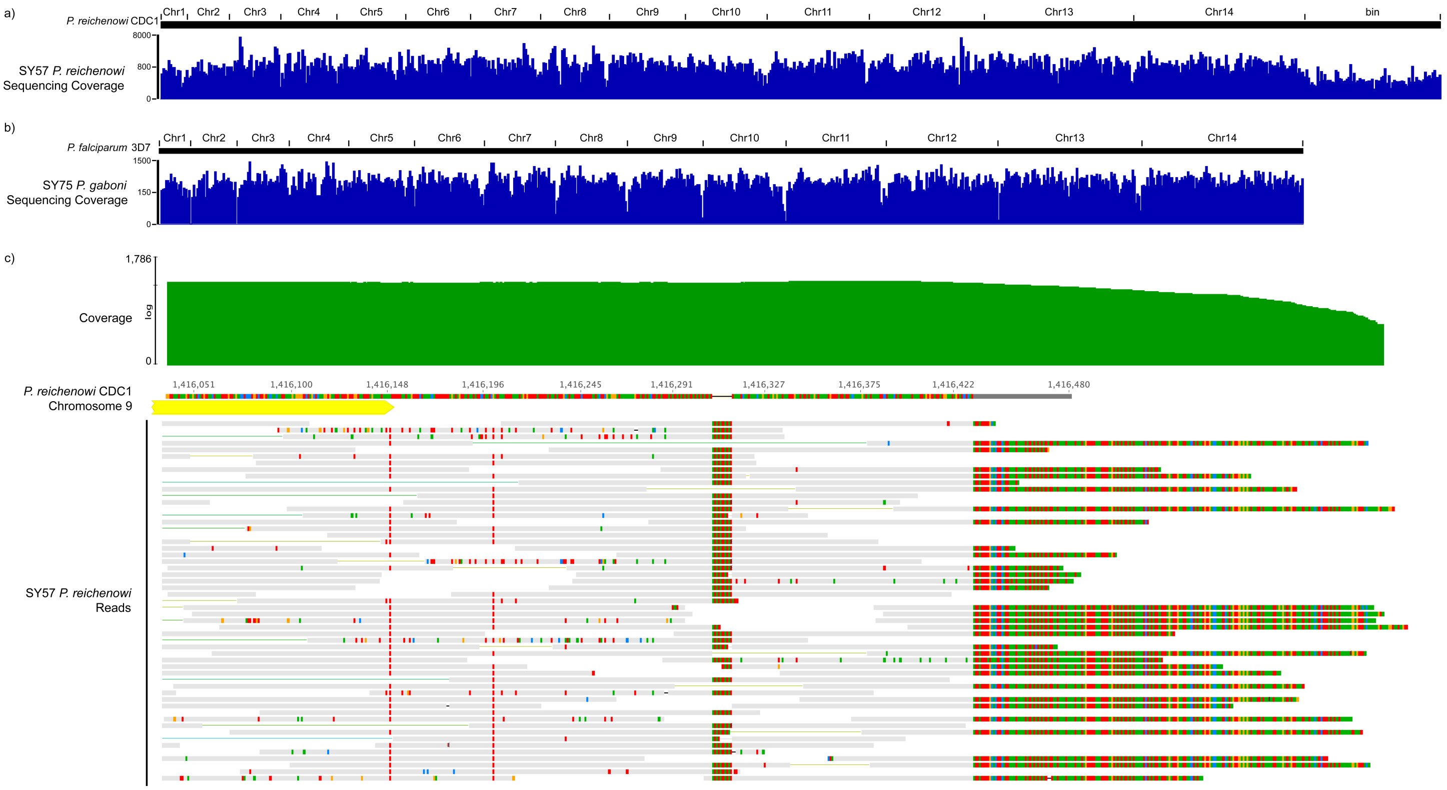

Supplementary Figure 3 Select amplification of *P. reichenowi* and *P. gaboni* genomes from unprocessed chimpanzee blood. (**a**) The MiSeq read depth is shown for all 14 chromosomes of the *P. reichenowi* strain SY57. Reads derived from 12 SWGA replicates were iteratively mapped to the PrCDC reference genome. (**b**) MiSeq read depth as in (**a**), but for the *P. gaboni* strain SY75. Reads derived from 6 SWGA replicates were iteratively mapped to the Pf3D7 reference genome. (**c**) Read coverage (green) in a sub-telomeric region of PrSY57. Individual reads (grey lines) are mapped to the end of the assembled PrCDC chromosome 9 indicated as a colored bar with base pair numbering. PrSY57 sequences that do not match this reference or extend beyond its 3' end (position 1,416,480) are highlighted in color (A, red; T, green; G, orange; C, blue). The SWGA-derived SY57 consensus sequence extends 220 bp beyond the reported 3' end of the PrCDC chromosome 9.

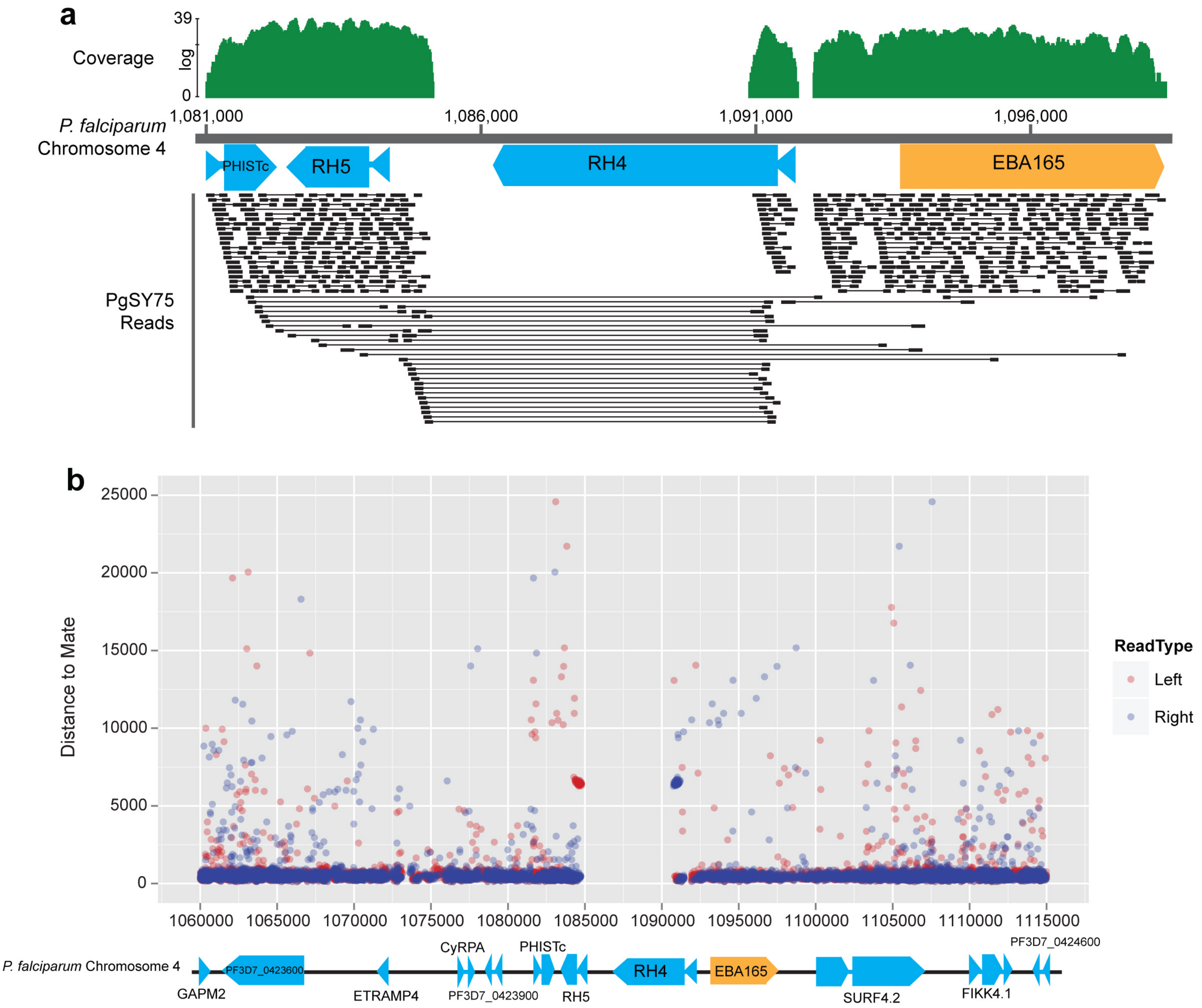
a
Coverage
39
log
0
P. falciparum
Chromosome 4
1,081,000
1,086,000
1,091,000
1,096,000
PHISTc
RH5
RH4
EBA165
PgSY75
Reads
b
Distance to Mate
25000
20000
15000
10000
5000
0
1060000
1065000
1070000
1075000
1080000
1085000
1090000
1095000
1100000
1105000
1110000
1115000
ReadType
Left
Right
P. falciparum Chromosome 4
GAPM2
PF3D7_0423600
ETRAMP4
CyRPA
PF3D7_0423900
PHISTc
RH5
RH4
EBA165
SURF4.2
FIKK4.1
PF3D7_0424600

Supplementary Figure 4 Gene loss in *P. gaboni* as determined by paired read mapping across a coverage gap. (**a**) A subset of PgSY75 read pairs is mapped to the *P. falciparum* 3D7 reference near the *RH4* gene on chromosome 4. Thick black lines denote individual reads, with coverage indicated in green. Thin lines connect reads that are paired, demonstrating the coverage gap. The positions of the Pf3D7 *PHISTc, RH5,* and *RH4* genes (blue) as well as the *EBA165* pseudogene (yellow) are shown, with their exon structure and direction of transcription indicated. (**b**) Scatter plot depicting the position of all PgSY75 paired reads on the Pf3D7 chromosome and their distance-to-mate near the RH4 locus. The leftmost read within a pair is shown in red, while the rightmost read is shown in blue. The gap in coverage indicates the absence of the second exon of *RH4* from *P. gaboni*. The position of genes (light blue) and the *EBA165* pseudogene (yellow) on Pf3D7 chromosome 4 is shown below.

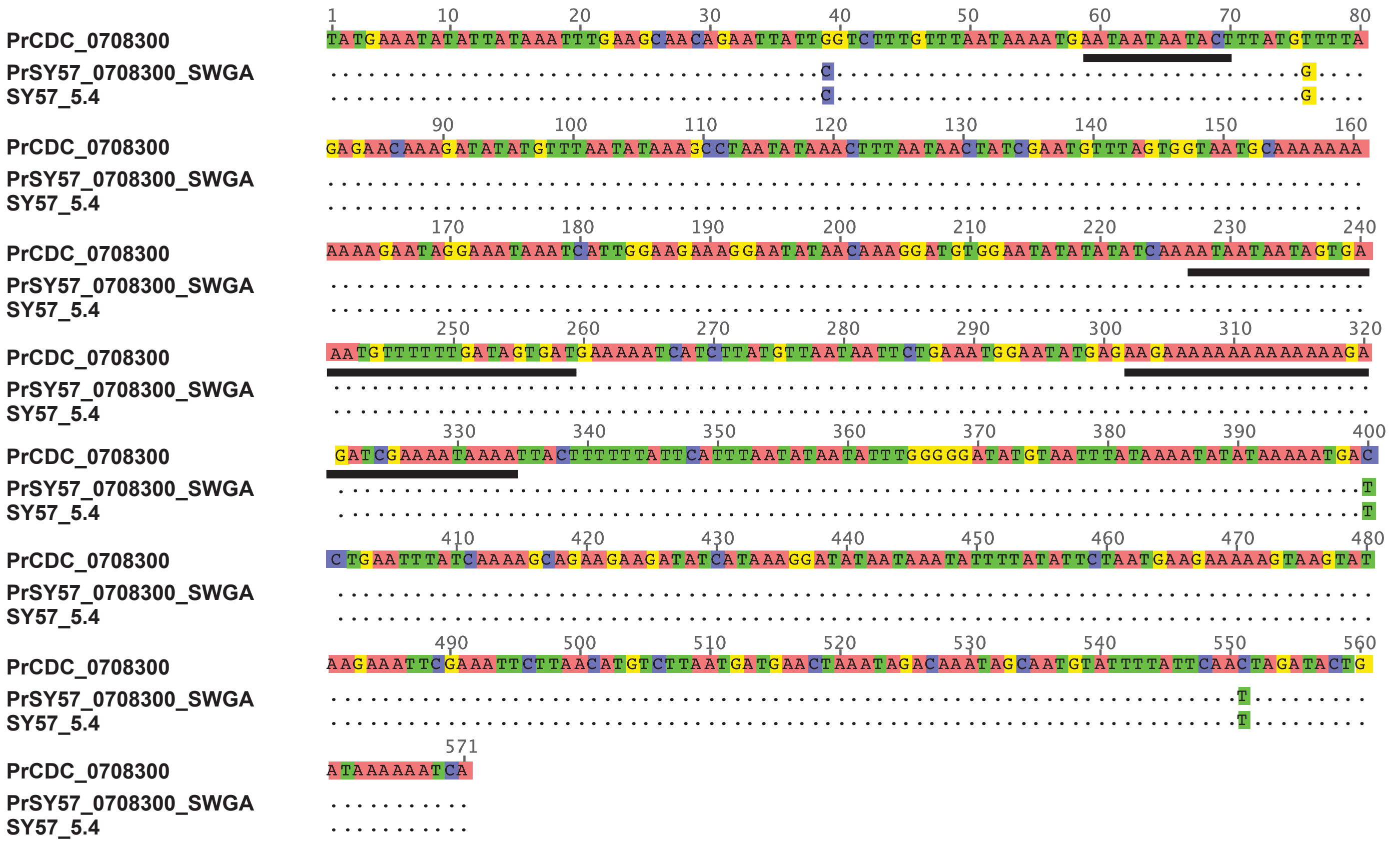
PrCDC_0708300
PrSY57_0708300_SWGA
SY57_5.4

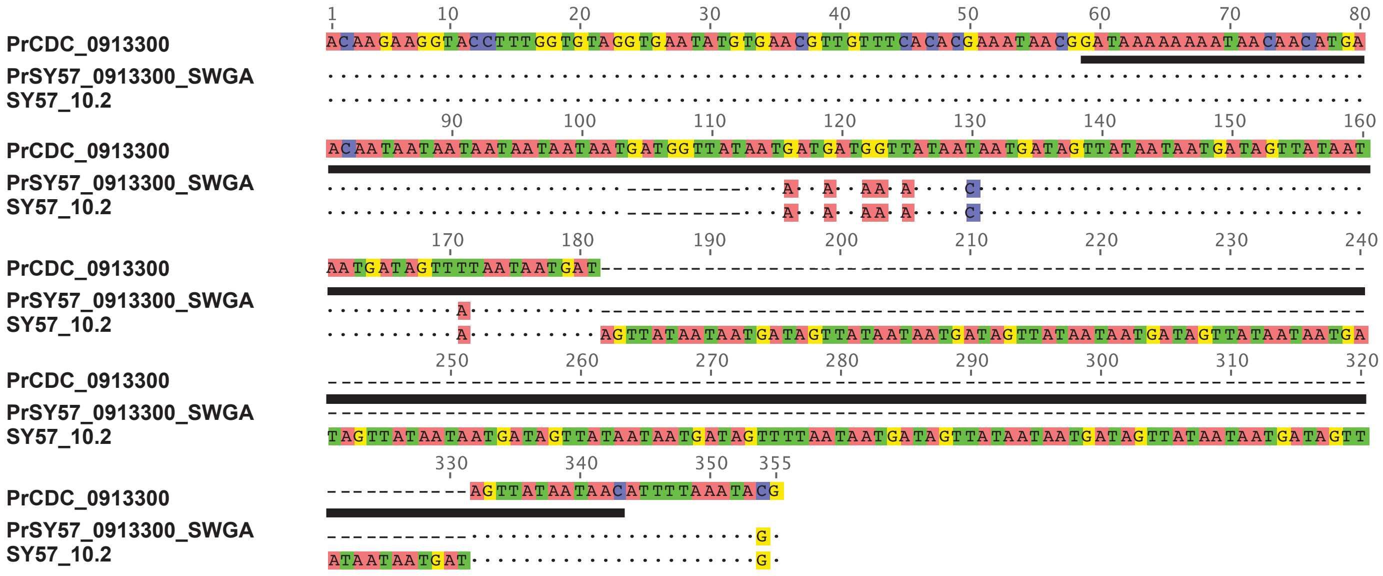
PrCDC_0913300
PrSY57_0913300_SWGA
SY57_10.2

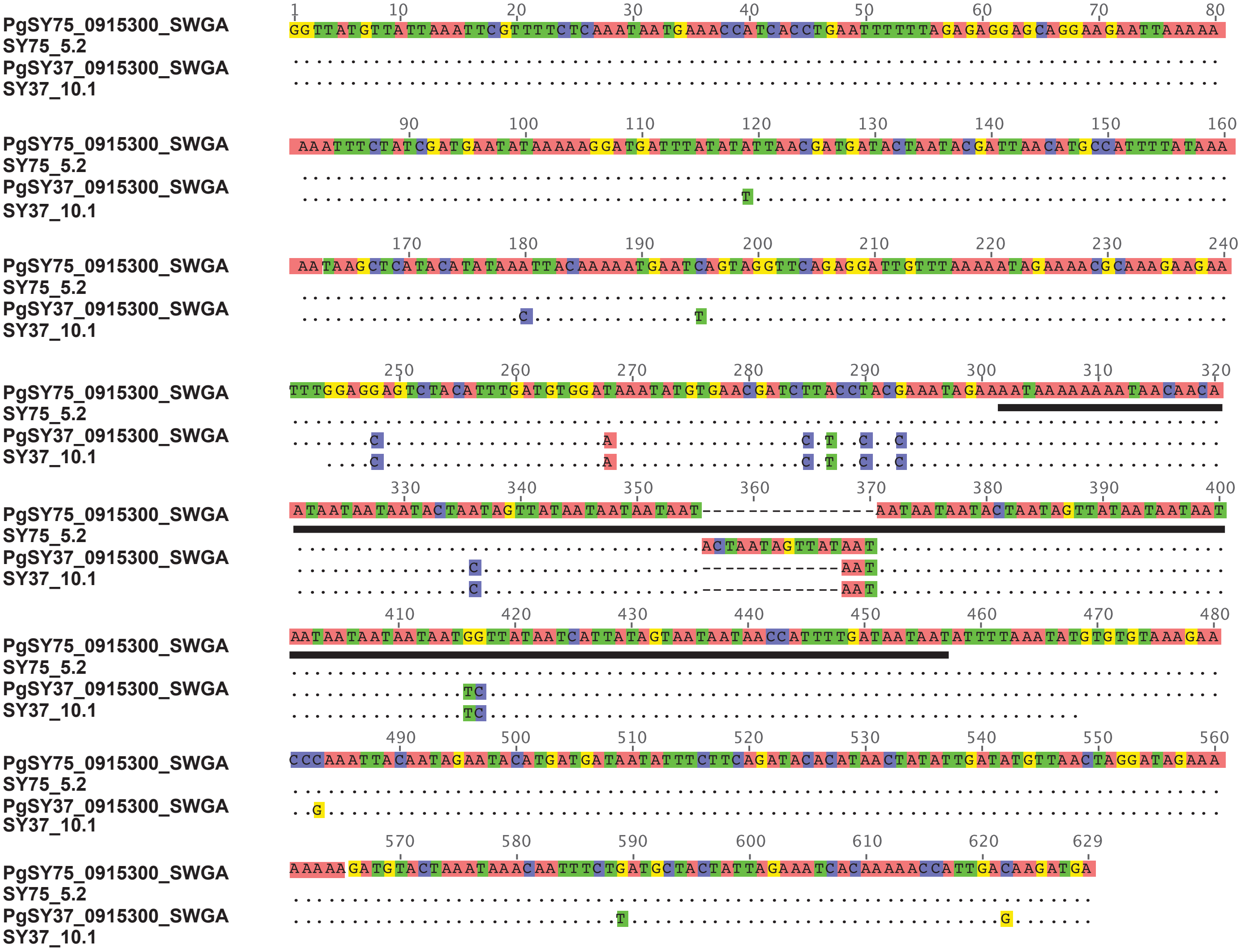
PgSY75_0915300_SWGA
SY75_5.2
PgSY37_0915300_SWGA
SY37_10.1

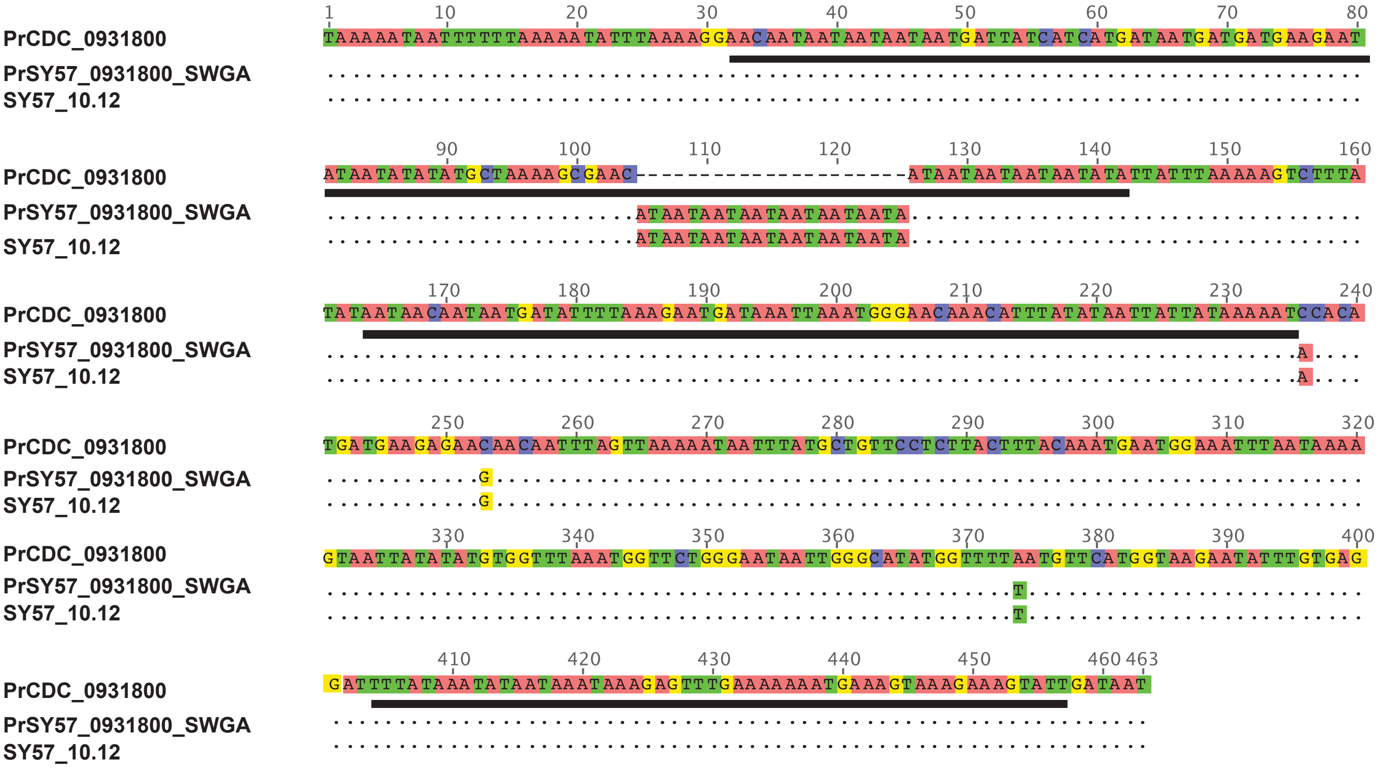
PrCDC_0931800
PrSY57_0931800_SWGA
SY57_10.12

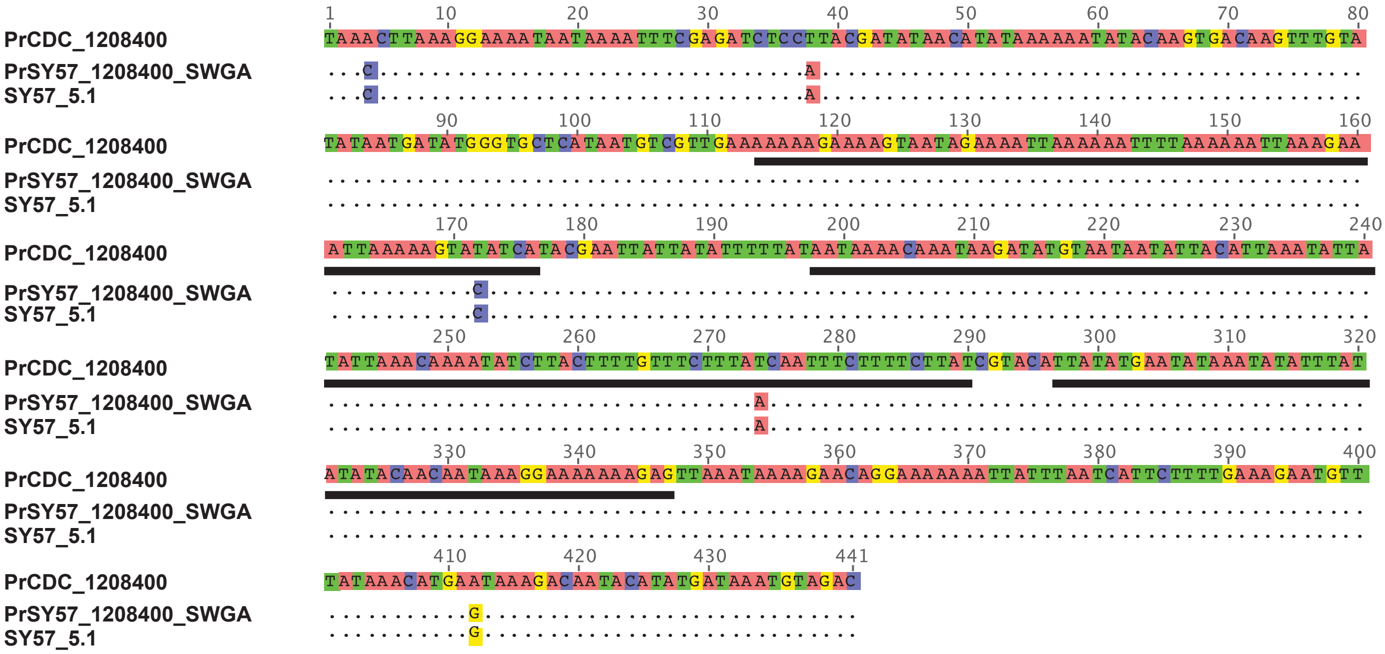
PrCDC_1208400
PrSY57_1208400_SWGA
SY57_5.1

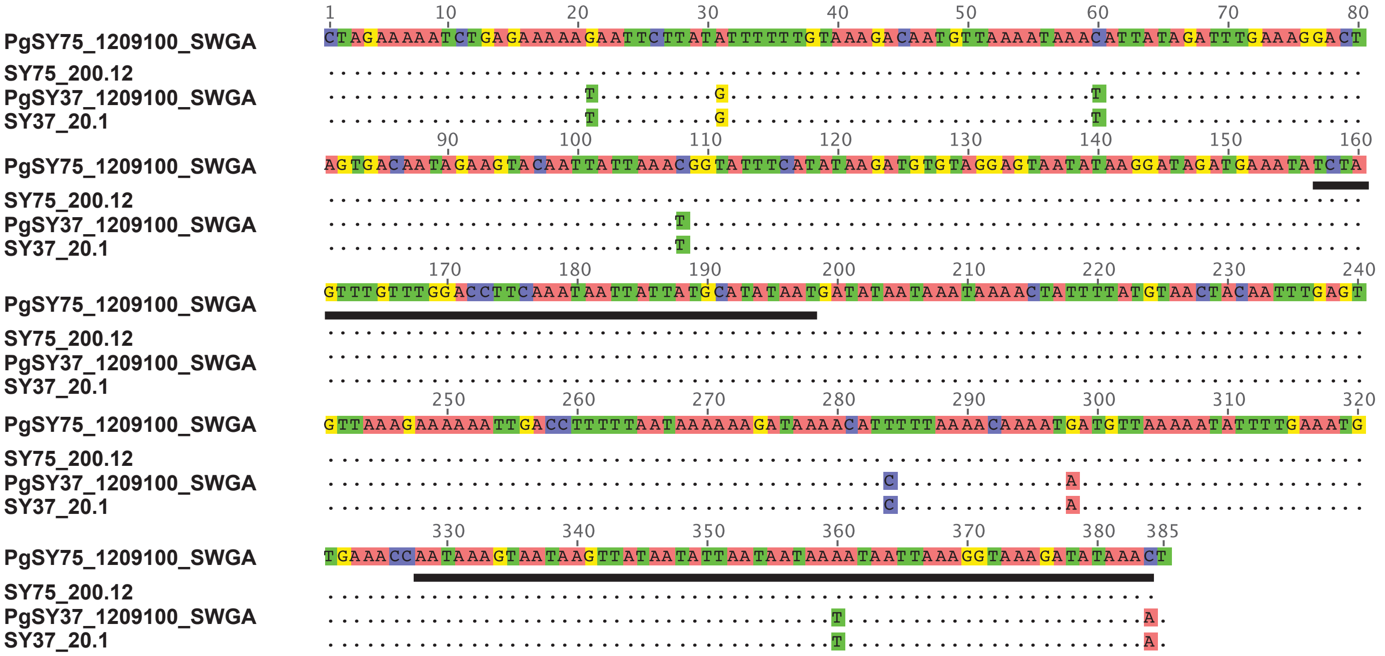
PgSY75_1209100_SWGA
SY75_200.12
PgSY37_1209100_SWGA
SY37_20.1

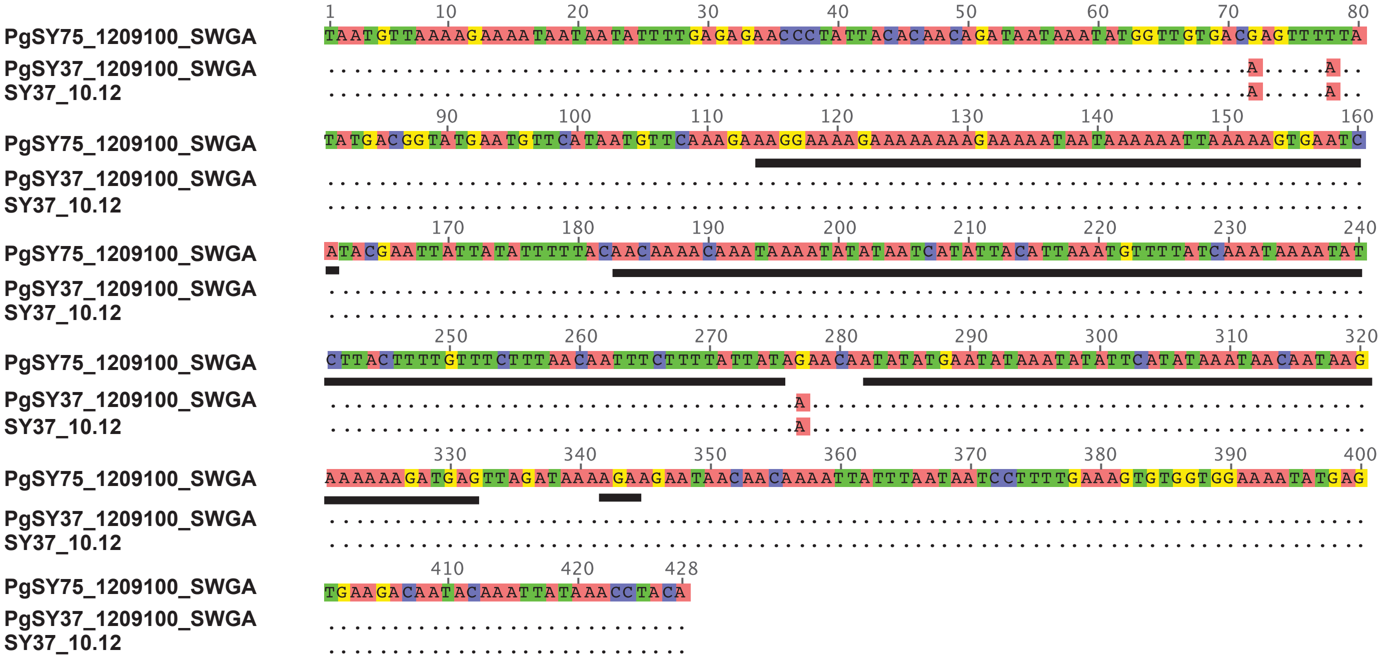
PgSY75_1209100_SWGA
PgSY37_1209100_SWGA
SY37_10.12

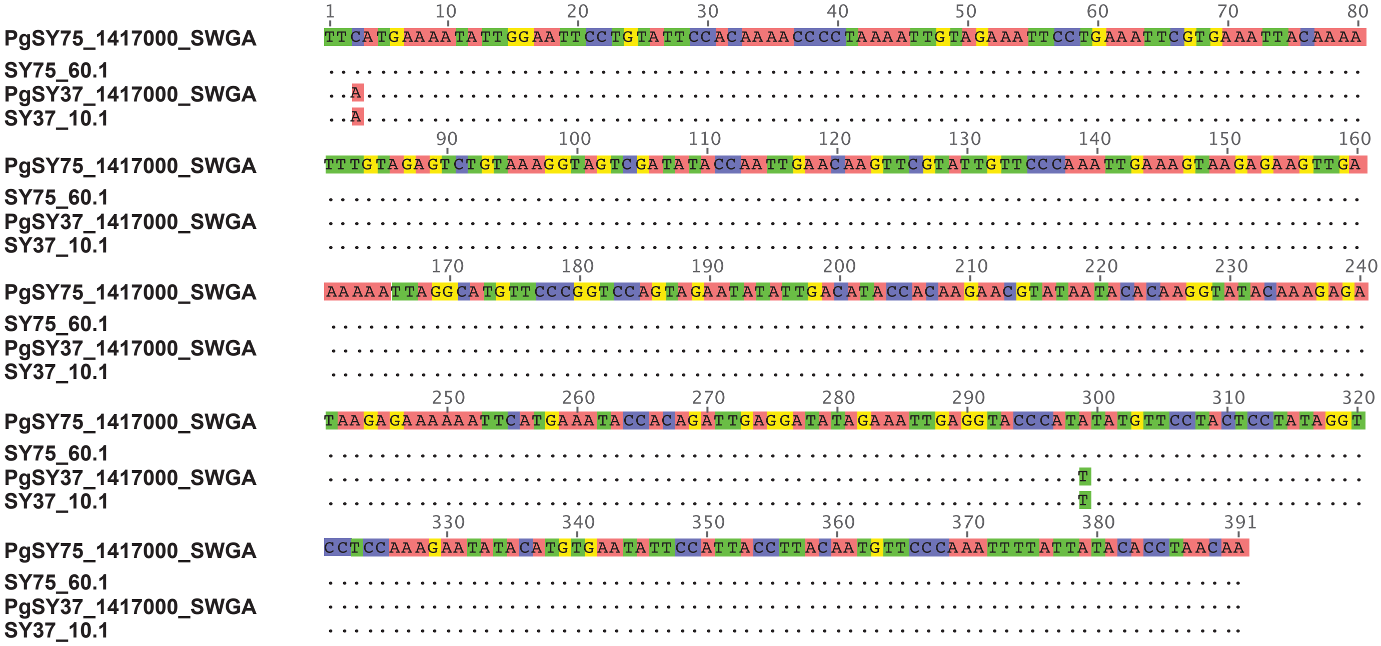
PgSY75_1417000_SWGA
SY75_60.1
PgSY37_1417000_SWGA
SY37_10.1

Supplementary Figure 5 Validation of SWGA-derived loci of high intra-species diversity. Genes exhibiting high within-species diversity in both *P. reichenowi* and *P. gaboni* (Supplementary Table 4) were subjected to limiting dilution PCR, and the resulting sequences were compared to the corresponding sequences in the respective SWGA-derived assembled genomes. Single template derived sequences are labeled to indicate their sample number, PCR dilution and well number (e.g., SY57_5.6 represents a single template derived sequence amplified from a 1:5 dilution of SY57 chimpanzee blood DNA and identified at position 6 in a plate of multiple PCR reactions). Alignments are shown for four *P. reichenowi* and three *P. gaboni* gene regions (indicated by gene ID), with PrCDC and PgSY75 used as reference sequences, respectively. Periods denote sequence identity, with gaps indicated by dashes introduced to optimize the alignment. Black lines indicate masked regions of low complexity that were excluded from diversity calculations (see Methods). SWGA and limiting dilution PCR derived sequences were identical in all regions, except for two indels in difficult-to-assemble regions.

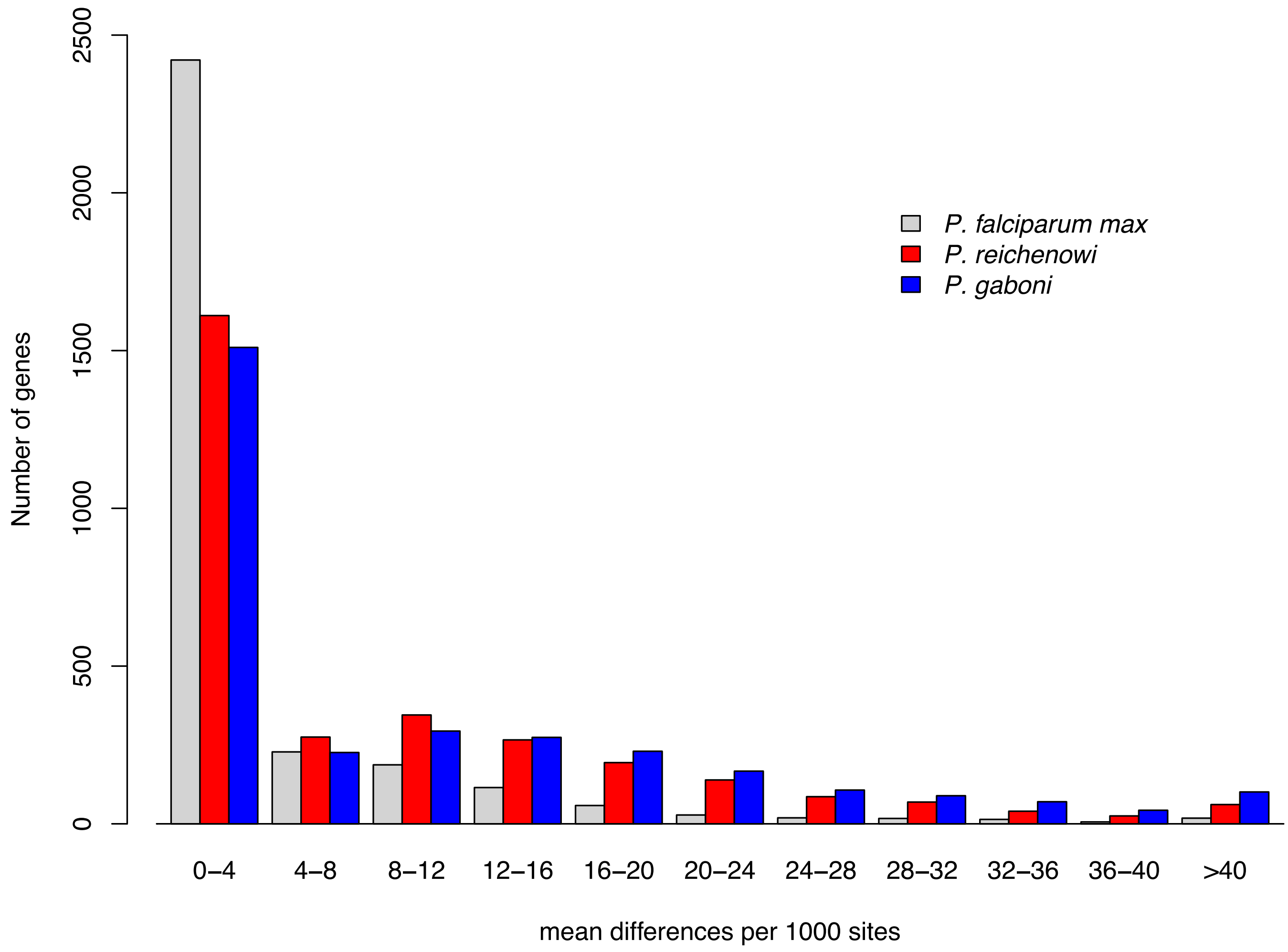

Supplementary Figure 6 Sequence diversity within three *Laverania* species. The maximum pairwise nucleotide sequence divergence was determined for 3,111 syntenic core genes at four-fold degenerate sites for 12 geographically diversity *P. falciparum* strains (grey), and then compared with the average nucleotide sequence diversity for two strains of *P. reichenowi* (red), and two strains of *P. gaboni* (blue), respectively. For *P. falciparum* field isolates, diversity information was obtained from SNP data (only datasets representing single parasite strains were used for analysis; see Methods for detail).

a

b

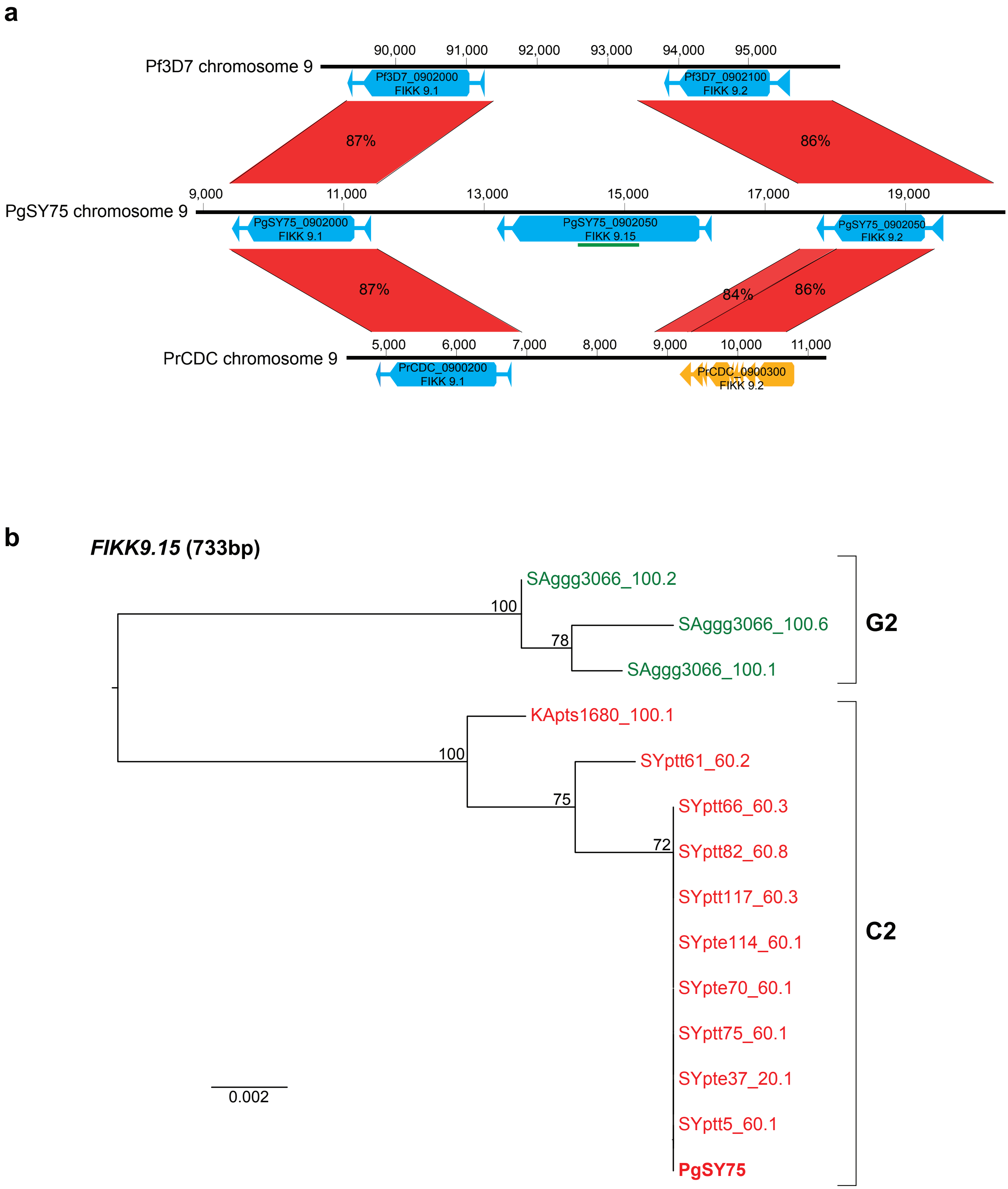

a
Pf3D7 chromosome 9
90,000
91,000
92,000
93,000
94,000
95,000
Pf3D7_0902000
FIKK 9.1
Pf3D7_0902100
FIKK 9.2
87%
86%
PgSY75 chromosome 9
9,000
11,000
13,000
15,000
17,000
19,000
PgSY75_0902000
FIKK 9.1
PgSY75_0902050
FIKK 9.15
PgSY75_0902050
FIKK 9.2
87%
84%
86%
PrCDC chromosome 9
5,000
6,000
7,000
8,000
9,000
10,000
11,000
PrCDC_0900200
FIKK 9.1
PrCDC_0900300
FIKK 9.2
b
FIKK9.15 (733bp)
SAggg3066_100.2
100
SAggg3066_100.6
78
SAggg3066_100.1
G2
KApts1680_100.1
100
SYptt61_60.2
75
SYptt66_60.3
72
SYptt82_60.8
SYptt117_60.3
SYpte114_60.1
SYpte70_60.1
SYptt75_60.1
SYpte37_20.1
SYptt5_60.1
PgSY75
C2
0.002

Supplementary Figure 7 Identification of *FIKK*9.15 orthologues in *P. gaboni* and *P. adleri*. (**a**) Genome organization of chromosome 9 surrounding the *FIKK9.15* locus in *P. falciparum* (top), *P. gaboni* (middle), and *P. reichenowi* (bottom). Black lines represent chromosomal sequences, with *FIKK*9.1 and *FIKK*9.2 genes (blue) and the *P. reichenowi FIKK*9.2 pseudogene (orange) drawn to scale. A green bar indicates the position of the PCR fragment analysed in (**b**). Red bars indicate regions of inter-species similarity with the percent sequence identity indicated. **(b)** Presence of *FIKK9.15* in *P. adleri*. A *FIKK*9.15 fragment (733 bp) was amplified by limiting dilution PCR from additional *P. gaboni* (C2) and *P. adleri* (G2) containing chimpanzee (red) and gorilla (green) samples (Supplementary Table 6). Sequences are color-coded, with capital letters indicating the field site as previously reported[1,2], except for one unknown location (SA) in Cameroon. Lower case letters denote the species and subspecies origin (ptt, *P. t. troglodytes*; pte, *P. t. ellioti*; pts, *P. t. schweinfurthii*; ggg, *G. g. gorilla*) of the samples. Single template PCR dilution (following the underscore) and well numbers (following the period) are also indicated. All sequences had intact *FIKK*9.15 reading frames. The PgSY75 genome sequence is bold faced. The tree was inferred using maximum likelihood methods[4], with numbers at internal nodes representing bootstrap support values[5]. The scale bar indicates 0.002 substitutions per site.

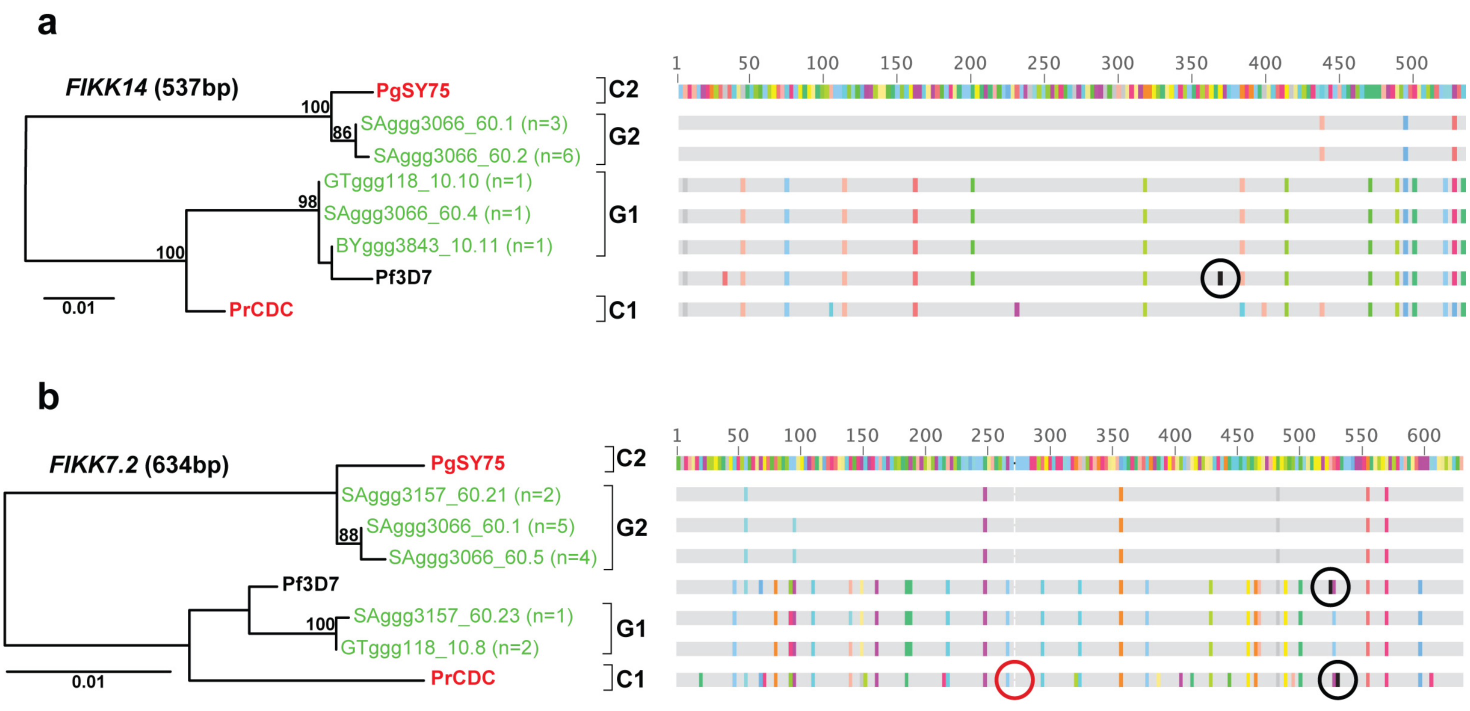

Supplementary Figure 8 Identification of *FIKK*7.2 and *FIKK*14 orthologues in *P. praefalciparum* and *P. adleri.* (**a**,**b**) Left panels: *FIKK*7.2 (634 bp) and *FIKK*14 (537bp) fragments were amplified by limiting dilution PCR from additional *P. praefalciparum* (G1) and *P. adleri* (G2) containing gorilla (green) samples (Supplementary Table 6). Sequences are labeled as in Supplementary Fig. 7, with the number of identical single template derived sequences shown in parentheses. *P. reichenowi* (PrCDC), *P. gaboni* (PgSY75) and *P. falciparum* (3D7) reference sequences are color coded (chimpanzee, red; human, black) and boldfaced. Phylogenetic trees were inferred using maximum likelihood methods[4], with bootstrap values greater than 70% listed[5]. The scale bar indicates 0.01 substitutions per site. (**a**,**b**) Right panels: Deduced protein sequences of the limiting dilution PCR derived fragments were aligned to the PgSY75 reference, with grey indicating sequence identity and colored stripes indicating amino acid mismatches. Black circles denote stop codons and a red circle indicates a frameshift mutation. All *P. praefalciparum* (G1) and *P. adleri* (G2) sequences had intact *FIKK*7.2 and *FIKK*14 reading frames.

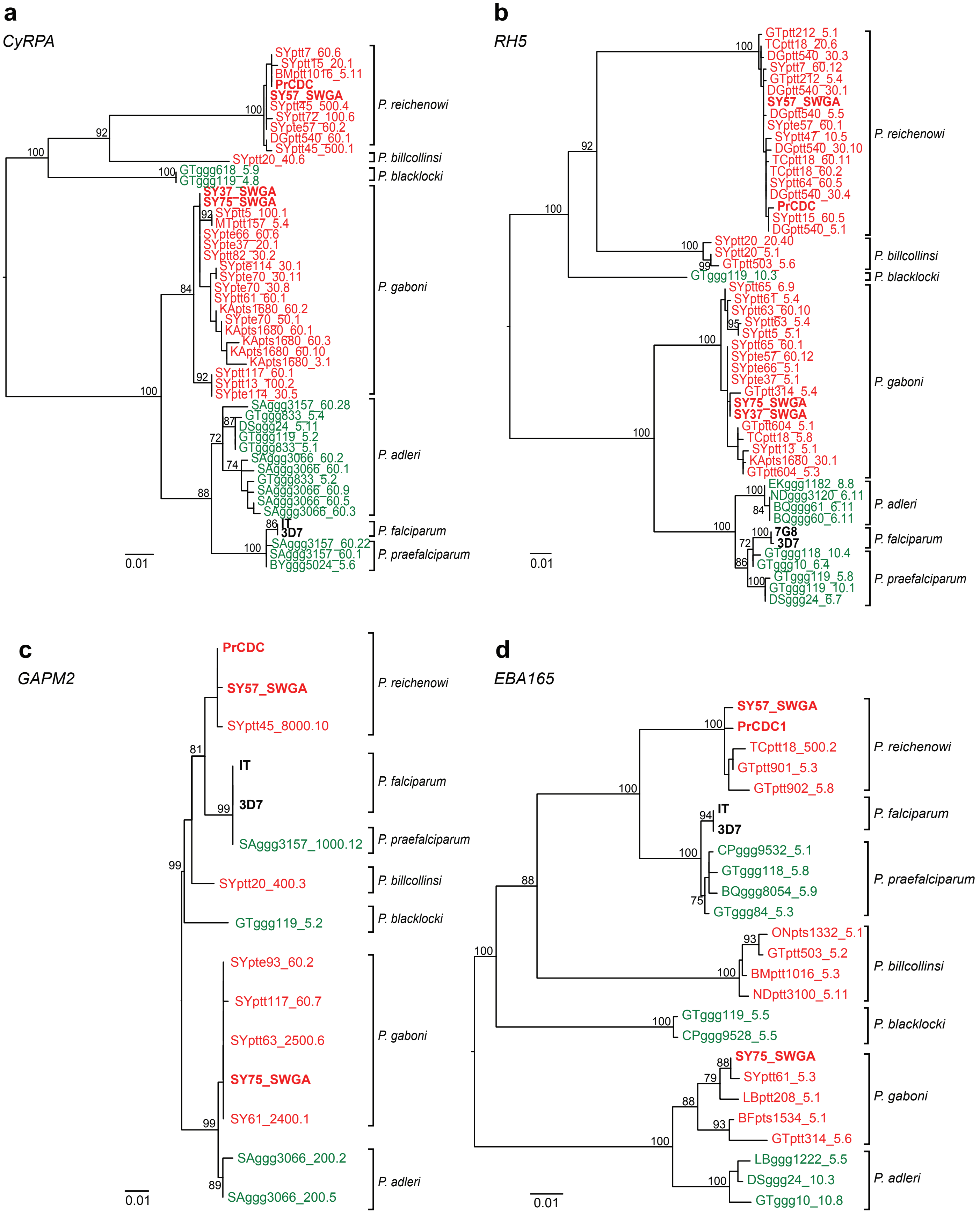
a
CyRPA
P. reichenowi
P. billcollinsi
P. blacklocki
P. gaboni
P. adleri
P. falciparum
P. praefalciparum
0.01
b
RH5
P. reichenowi
P. billcollinsi
P. blacklocki
P. gaboni
P. adleri
P. falciparum
P. praefalciparum
0.01
c
GAPM2
PrCDC
SY57_SWGA
SYptt45_8000.10
P. reichenowi
IT
3D7
P. falciparum
SAggg3157_1000.12
P. praefalciparum
SYptt20_400.3
P. billcollinsi
GTggg119_5.2
P. blacklocki
SYpte93_60.2
SYptt117_60.7
SYptt63_2500.6
SY75_SWGA
SY61_2400.1
P. gaboni
SAggg3066_200.2
SAggg3066_200.5
P. adleri
0.01
d
EBA165
SY57_SWGA
PrCDC1
TCptt18_500.2
GTptt901_5.3
GTptt902_5.8
P. reichenowi
IT
3D7
P. falciparum
CPggg9532_5.1
GTggg118_5.8
BQggg8054_5.9
GTggg84_5.3
P. praefalciparum
ONpts1332_5.1
GTptt503_5.2
BMptt1016_5.3
NDptt3100_5.11
P. billcollinsi
GTggg119_5.5
CPggg9528_5.5
P. blacklocki
SY75_SWGA
SYptt61_5.3
LBptt208_5.1
BFpts1534_5.1
GTptt314_5.6
P. gaboni
LBggg1222_5.5
DSggg24_10.3
GTggg10_10.8
P. adleri
0.01

Supplementary Figure 9 Phylogenetic relationships of *Laverania* species in genes within and outside the horizontally transferred genome segment. (**a**) *RH5* (806 bp), (**b**) *CyRPA* (790 bp), (**c**) *GAPM2* (457 bp), and (**d**) *EBA165* (790 bp) gene sequences were amplified by limiting dilution PCR from additional chimpanzee (red) and gorilla (green) samples (Supplementary Table 6) or obtained from reference genome assemblies (Pf3D7, PfIT, Pf7G8, PrCDC, PgSY75, PgSY37, and PrSY57). Sequences are labeled as in Supplementary Fig. 7, except for one sample from the Tchimpounga (TC) sanctuary in the Republic of Congo. (**a,b**) Trees of *CyRPA* and *RH5* sequences reveal an unexpectedly close relationship of the *P. falciparum*/*P. praefalciparum* clade with the gorilla parasite *P. adleri*, consistent with the transfer of genetic material from an ancestor of *P. adleri* to an ancestor of *P. praefalciparum*. (**c,d**) In contrast, trees derived from *GAPM2* and *EBA165* sequences are consistent with previous *Laverania* topologies[1]. Trees were generated using maximum likelihood phylogenetic methods[4], with numbers on branches indicating percent bootstrap support[5]. The scale bars indicate 0.01 substitutions per site.

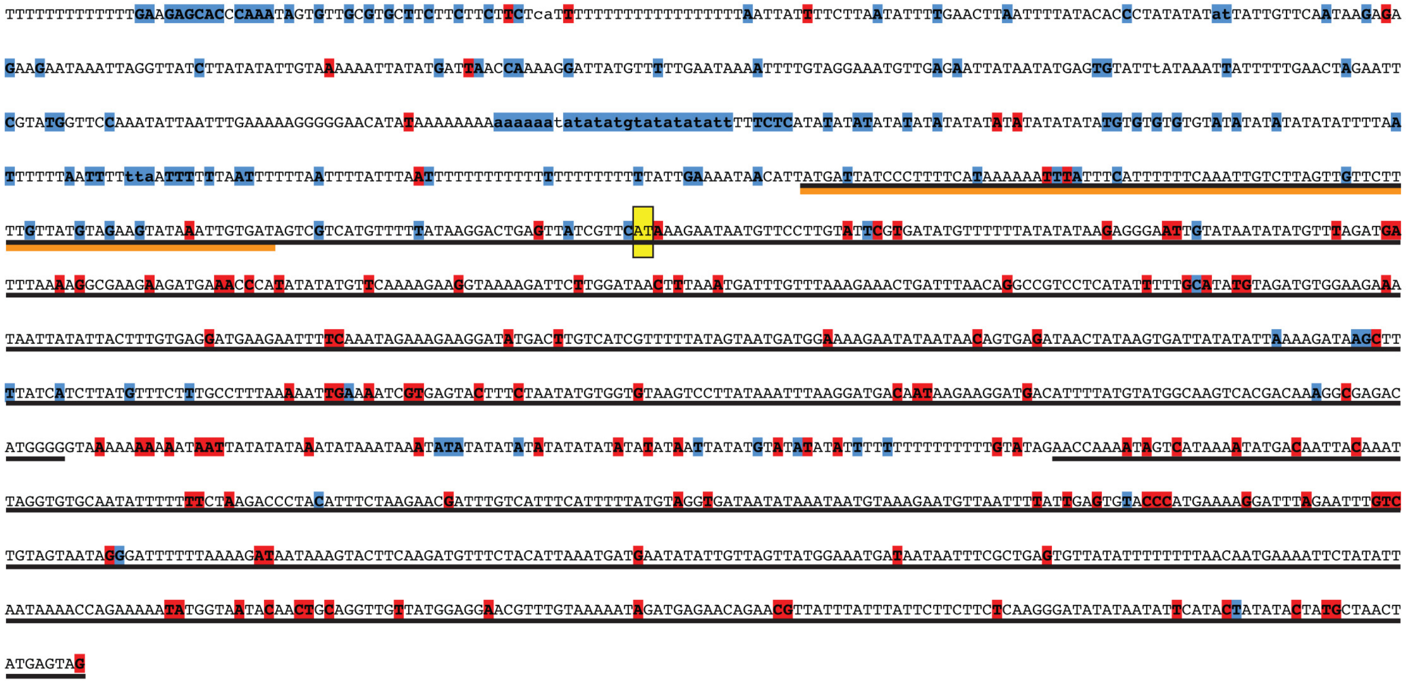

Supplementary Figure 10 Position of the 5' breakpoint of the transferred fragment within the *P. falciparum CyRPA* gene. The *P. falciparum* (3D7) sequence is shown, with nucleotides shared only with *P. reichenowi* (CDC) or only with *P. gaboni* (SY75) highlighted in blue and red, respectively. Lowercase bases indicate an insertion in *P. falciparum* with respect to either *P. reichenowi* or *P. gaboni*. The yellow box indicates the approximate breakpoint of the transferred fragment, as determined by a maximum chi squared method[6]. The two exons of *CyRPA* are underlined in black, with the bases encoding the predicted signal sequence underlined in orange.

Supplementary Table 1 Selective whole genome amplification of *P. reichenowi* and *P. gaboni* from unprocessed chimpanzee blood samples

Laverania species composition[b]										MiSeq sequencing of SWGA products						
**Sample[a]**	***cytB***	***EBA165***	***EBA175***	***p47***	***p48/45***	***ldh***	***RH5***	***clpM***	**Total**	**Percent *Plasmodium* DNA[c]**	**SWGA replicates**	**MiSeq reads**	**Percent reads mapping to chimpanzee genome**	**Percent reads mapping to *Plasmodium* reference[d]**	**Percent unmapped reads**	**Percent read coverage ≥5x**
SYpte57	C1 (92)	C1 (6)	C1 (3)	C1 (7)	C1 (7)	C1 (32)	C1 (9)<br>C2 (1)	C1 (1)	C1 (157)<br>C2 (1)	0.0054	12	27,038,372	2.4	88.8	8.8	95.8
SYptt75	C2 (15)	C2 (5)	C2 (3)	C2 (53)	C2 (8)	C2 (29)	nd[e]	C2 (9)	C2 (122)	0.14	6	31,445,752	0.1	73.2	26.7	78.8
SYpte37	C2 (8)	C2 (3)	C2 (6)	C2 (5)	C2 (4)	C2 (27)	C2 (3)	C2 (3)	C2 (59)	0.00081	16	39,615,918	0.7	61.2	38.1	68.5

[a]Whole blood samples were collected for veterinary purposes from captive chimpanzees (*Pan troglodytes*) housed at the Sanaga Yong Chimpanzee Rescue Center (SY) in Cameroon. Ape species and subspecies were determined by mitochondrial DNA analysis (*ptt, P. t. troglodytes; pte, P. t. ellioti*) as described[1].

[b]The *Laverania* species composition of each chimpanzee blood sample was determined by limiting dilution (single template) PCR of mitochondrial (*cytB*), nuclear (*EBA165, EBA175, p47, p48/45, ldh, RH5*) and apicoplast (*clpM* gene encoding the Clp chaperone PfC10_API0060; previously called *clpC*[1]) gene fragments as described[1]. C1, *P. reichenowi*; C2, *P. gaboni*. Brackets indicate the number of single template-derived sequences obtained for each *Laverania* species.

[c]Determined by real time PCR of total blood DNA.

[d]PrSY57 reads were mapped to the PrCDC reference genome; PgSY75 and PgSY37 reads were mapped to the Pf3D7 reference genome.

[e]nd, not done.

Supplementary Table 2 Gene loss and acquisition in *Laverania* species

Description	Pf3D7 Locus ID	PrCDC Locus ID	PgSY75 Locus ID	*P. falciparum*	*P. reichenowi*	*P. gaboni*	Evidence
Serine/threonine protein kinase, FIKK family	n/a[a]	n/a	PGSY75_0902050	gene loss	gene loss	intact gene	Long branch in Fig. 3 indicates origin prior to *Laverania* radiation
Endonuclease/exonuclease/phosphatase domain containing protein	n/a	n/a	PGSY75_0905950	gene loss	gene loss	intact gene	Syntenic orthologues present in non-*Laverania* parasites
Reticulocyte binding protein homologue 7 (RH7)	n/a	PRCDC_1334400	PGSY75_0019600A-C	gene loss	intact gene	incomplete gene[b]	Parsimony
Serine repeat antigen 3 (SERA3)	PF3D7_0207800	n/a	PGSY75_0001300	intact gene	gene loss	incomplete gene	Parsimony
Reticulocyte binding protein homologue 4 (RH4)	PF3D7_0424200	PRCDC_0421400	n/a	intact gene	intact gene	gene fragment	See Supplementary Fig. 4
conserved *Plasmodium* protein, unknown function	PF3D7_0917400	PRCDC_0915400	n/a	intact gene	intact gene	gene loss	Syntenic orthologues present in non-*Laverania* parasites
Reticulocyte binding protein homologue 2a (RH2a)	PF3D7_1335400	n/a	n/a	intact gene (duplication)	absent	absent	Parsimony

[a]n/a, not available.
[b]gene assembly incomplete

Supplementary Table 3 Pseudogenes in *Laverania* Species

Description	Pf3D7 ID	PrCDC ID	PgSY75 ID	*P. falciparum*[a]	*P. reichenowi*[b]	*P. gaboni*[c]
Lysophospholipase, putative	PF3D7_0102400	PRCDC_0100300	PGSY75_0102400	intact gene/pseudogene[d]	intact gene	ambiguous[e]
MC-2TM Maurer's cleft two transmembrane protein (MC-2TM)	PF3D7_0114100	PRCDC_0112300	PGSY75_0114100	intact gene	intact gene	pseudogene
Exported protein, unknown function	PF3D7_0114300	PRCDC_0112500	n/a	pseudogene	intact gene	unassembled[f]
Pentafunctional AROM polypeptide, putative (AROM)	PF3D7_0206300	PRCDC_0205400	PGSY75_0206300	pseudogene	ambiguous	ambiguous
DnaJ protein, putative	PF3D7_0220400	PRCDC_0219300	PGSY75_0220400	pseudogene	intact gene	intact gene
Exported protein (hyp9), unknown function	PF3D7_0220700	PRCDC_0219600	PGSY75_0220700	intact gene	intact gene	pseudogene
Exported protein, unknown function	PF3D7_0221100	PRCDC_0219800	n/a	pseudogene	pseudogene	unassembled
Conserved *Plasmodium* protein, unknown function	PF3D7_0315600	PRCDC_0314900	PGSY75_0315600	intact gene	intact gene	pseudogene
RESA-like protein	PF3D7_0324000	PRCDC_0323300	n/a	intact gene	pseudogene	unassembled
Plasmodium exported protein, unknown function	PF3D7_0401700	PRCDC_0004800	n/a	pseudogene	intact gene	unassembled
Surface-associated interspersed protein 4.1 (SURFIN 4.1)	PF3D7_0402200	PRCDC_0005300	PGSY75_0402200	intact gene/pseudogene	intact gene	ambiguous
Reticulocyte binding protein homologue 1 (RH1)	PF3D7_0402300	PRCDC_0005400	PGSY75_0402200	intact gene	pseudogene	intact gene
Erythrocyte binding antigen-165 (EBA165)	PF3D7_0424300	PRCDC_0421500	PGSY75_0424300	pseudogene	intact gene	intact gene
Exported protein (PHISTb), unknown function	PF3D7_0424800	PRCDC_0422000	PGSY75_0424800	intact gene	pseudogene	intact gene
Histone deacetylase, putative	PF3D7_0506600	PRCDC_0505800	PGSY75_0506600	pseudogene	pseudogene	ambiguous
Conserved *Plasmodium* protein, unknown function	PF3D7_0512450	PRCDC_0511650	PGSY75_0512450	pseudogene	pseudogene	pseudogene
Exported protein, unknown function	PF3D7_0532600	PRCDC_0531600	n/a	intact gene	pseudogene	unassembled
Exported protein (PHISTa), unknown function	PF3D7_0601700	PRCDC_0600300	n/a	pseudogene	pseudogene	unassembled
Conserved *Plasmodium* protein, unknown function	PF3D7_0624800	PRCDC_0623200	PGSY75_0624800	intact gene	intact gene/pseudogene	pseudogene
Plasmodium exported protein (PHISTb), unknown function	PF3D7_0631100	PRCDC_0629500	PGSY75_0631100	intact gene	ambiguous	pseudogene
Plasmodium exported protein (PHISTb), unknown function	PF3D7_0702100	PRCDC_0700300	PGSY75_0702100	pseudogene	intact gene	intact gene
Lysophospholipase, putative	PF3D7_0702200	PRCDC_0700400	PGSY75_0702200	intact gene	pseudogene	intact gene
Serine/threonine protein kinase, FIKK family (FIKK7.2)	PF3D7_0731400	PRCDC_0728200	PGSY75_0731400	pseudogene	pseudogene	intact gene
Plasmodium exported protein (hyp9), unknown function	PF3D7_0731700	PRCDC_0728500	PGSY75_0731700	intact gene	intact gene	pseudogene

Plasmodium exported protein (hyp7), unknown function	PF3D7_0800900	PRCDC_0800500	PGSY75_0006100	pseudogene	intact gene	intact gene
Surface-associated interspersed protein 8.1 (SURFIN 8.1)	PF3D7_0831100	PRCDC_0830400	PGSY75_0831100	intact gene	pseudogene	intact gene
Plasmodium exported protein (PHISTa), unknown function	PF3D7_0831750	PRCDC_0831000	n/a	pseudogene	pseudogene	unassembled
Serine/threonine protein kinase, FIKK family (FIKK9.2)	PF3D7_0902100	PRCDC_0900300	PGSY75_0902100	intact gene	pseudogene	intact gene
Serine/threonine protein kinase, FIKK family (FIKK9.5)	PF3D7_0902400	PRCDC_0900600	PGSY75_0008500	intact gene	pseudogene	pseudogene
Plasmodium exported protein, unknown function	PF3D7_0935700	PRCDC_0933700	PGSY75_0935700	intact gene	pseudogene	intact gene
Plasmodium exported protein, unknown function	PF3D7_0936500	PRCDC_0934600	PGSY75_0936500	intact gene	pseudogene	intact gene
Plasmodium exported protein (PHISTb), unknown function	PF3D7_0936900	PRCDC_0935000	PGSY75_0936900	pseudogene	pseudogene	ambiguous
Conserved *Plasmodium* protein, unknown function	PF3D7_1008300	PRCDC_1007700	n/a	pseudogene	pseudogene	gene loss[g]
Conserved *Plasmodium* protein, unknown function	PF3D7_1029000	PRCDC_1028300	PGSY75_1029000	pseudogene	pseudogene	intact gene
Merozoite surface protein (MSP3.8)	PF3D7_1036300	PRCDC_1035400	n/a	intact gene	pseudogene	unassembled
Gametocyte-specific protein	PF3D7_1038400	PRCDC_1037500	n/a	intact gene	pseudogene	unassembled
Plasmodium exported protein, unknown function	PF3D7_1038700	PRCDC_1037700	PGSY75_1038700	intact gene	pseudogene	intact gene
Plasmodium exported protein, unknown function	PF3D7_1102100	PRCDC_1100600	n/a	pseudogene	intact gene	unassembled
DnaJ protein, putative	PF3D7_1102200	PRCDC_1100700	PGSY75_1102200	intact gene	intact gene	pseudogene
Plasmodium exported protein (hyp11), unknown function	PF3D7_1102900	PRCDC_1101400	PGSY75_1102900	intact gene	pseudogene	intact gene
Conserved *Plasmodium* protein, unknown function	PF3D7_1127800	PRCDC_1126300	PGSY75_1127800	intact gene	intact gene	pseudogene
UNC-50 protein, putative	PF3D7_1129850	PRCDC_1128250	n/a	pseudogene	pseudogene	gene loss
Conserved *Plasmodium* protein, unknown function	PF3D7_1135200	PRCDC_1133600	PGSY75_1135200	pseudogene	pseudogene	pseudogene
Ring-infected erythrocyte surface antigen 2 (RESA2)	PF3D7_1149500	PRCDC_1147400	PGSY75_1149500	pseudogene	intact gene	intact gene
Plasmodium exported protein (PHISTb), unknown function	PF3D7_1201000	PRCDC_1200400	PGSY75_1201000	intact gene	pseudogene	intact gene
Conserved *Plasmodium* protein, unknown function	PF3D7_1220200	PRCDC_1219500	PGSY75_1220200	pseudogene	pseudogene	pseudogene
Vacuolar transporter chaperone, putative	PF3D7_1230200	PRCDC_1229500	PGSY75_1230200	pseudogene	pseudogene	ambiguous
Conserved *Plasmodium* protein, unknown function	PF3D7_1234200	PRCDC_1233400	PGSY75_1234200	pseudogene	pseudogene	intact gene
Reticulocyte binding protein homologue 3 (RH3)	PF3D7_1252400	PRCDC_1251800	PGSY75_1252400	pseudogene	intact gene	ambiguous

Exported protein (PHISTa-like), unknown function	PF3D7_1301300	PRCDC_1300200	n/a	pseudogene	pseudogene	unassembled
MSP7-like protein (MSRP3)	PF3D7_1334600	PRCDC_1333600	PGSY75_0019100	intact gene	pseudogene	ambiguous
MSP7-like protein, fragment	PF3D7_1334900	PRCDC_1333900	n/a	pseudogene	pseudogene	unassembled
Reticulocyte binding protein homologue 6 (RH6)	PF3D7_1335200	PRCDC_1334300	PGSY75_0019400	pseudogene	pseudogene	ambiguous
Conserved *Plasmodium* membrane protein, unknown function	PF3D7_1351300	PRCDC_1350300	PGSY75_1351300	intact gene	intact gene	pseudogene
Tryptophan-rich antigen	PF3D7_1353000	PRCDC_1352000	PGSY75_1353000	pseudogene	intact gene	ambiguous
Conserved *Plasmodium* protein, unknown function	PF3D7_1364700	PRCDC_1363600	PGSY75_1364700	pseudogene	pseudogene	pseudogene
Erythrocyte binding like protein 1, fragment	PF3D7_1371600	PRCDC_1369500	PGSY75_0022200A and B	pseudogene	pseudogene	ambiguous
Serine/threonine protein kinase, FIKK family	PF3D7_1371700	PRCDC_1369600	PGSY75_1371700	intact gene	intact gene	pseudogene
Plasmodium exported protein, unknown function	PF3D7_1371900	PRCDC_1369800	PGSY75_1371900	intact gene	pseudogene	intact gene
Plasmodium exported protein, unknown function	PF3D7_1401700	PRCDC_1401000	PGSY75_0022600	intact gene	intact gene	pseudogene
Cyclic nucleotide-binding protein, putative (cNBP)	PF3D7_1417400	PRCDC_1416700	PGSY75_1417400	intact gene/pseudogene	intact gene	intact gene
Apyrase, putative	PF3D7_1431800	PRCDC_1431100	PGSY75_1431800	intact gene	pseudogene	intact gene
Protein kinase, putative	PF3D7_1433900	PRCDC_1433200	PGSY75_1433900	intact gene	intact gene	pseudogene
Conserved *Plasmodium* protein, unknown function	PF3D7_1463100	PRCDC_1462350	PGSY75_1463100	pseudogene	pseudogene	pseudogene
Serine/threonine protein kinase, FIKK family (FIKK14)	PF3D7_1476400	PRCDC_1475400	PGSY75_1476400	pseudogene	intact gene	intact gene
Plasmodium exported protein, unknown function[h]	PF3D7_1476900	PRCDC_1475900	PGSY75_0025600	pseudogene	pseudogene	pseudogene
Plasmodium exported protein (hyp17), unknown function	PF3D7_1477000	PRCDC_1476000	n/a	intact gene/pseudogene	intact gene	unassembled
Plasmodium exported protein (hyp15), unknown function	PF3D7_1477200	PRCDC_1476200	PGSY75_0025700	intact gene	pseudogene	intact gene
Acyl-CoA synthetase (ACS1b)	PF3D7_1477900	PRCDC_1476900	PGSY75_1477900	pseudogene	intact gene	intact gene
Plasmodium exported protein (PHISTa), unknown function	PF3D7_1478000	PRCDC_1477000	n/a	intact gene	pseudogene	unassembled
Plasmodium exported protein, unknown function	PF3D7_1478200	PRCDC_1477200	n/a	pseudogene	pseudogene	unassembled
Plasmodium exported protein, unknown function	PF3D7_1478300	PRCDC_1477300	PGSY75_0026500	pseudogene	pseudogene	intact gene
Plasmodium exported protein, unknown function	PF3D7_1478400	PRCDC_1477400	PGSY75_1478400	intact gene/pseudogene	intact gene	intact gene
Plasmodium exported protein (PHISTa), unknown function	PF3D7_1478500	PRCDC_1477500	n/a	pseudogene	pseudogene	unassembled

Plasmodium exported protein, unknown function	PF3D7_1478700	PRCDC_1477700	n/a	pseudogene	intact gene	unassembled
Cytoadherence linked asexual protein 9, pseudogene	n/a	PRCDC_0933800	n/a	gene loss	pseudogene	unassembled

[a]Pf3D7 and PfIT genomes were used in the analysis.
[b]PrCDC and PrSY57 genomes were used in the analysis.
[c]only the PgSY75 genome was used in the analysis.
[d]Gene is a pseudogene in some, but not all, strains analysed.
[e]Gene was incomplete due to sequence gaps, but otherwise intact.
[f]Gene could not be analysed due to a gap in the assembly, but mapping of reads to the reference indicated an orthologue was likely present.
[g]Gene absent in this species; gene loss inferred by parsimony and/or presence of a syntenic orthologue in non-*Laverania Plasmodium* species.
[h]Gene models differ between Pf3D7 and PrCDC; since the Pf3D7 model is likely more accurate, this has been used for all three species.

Supplementary Table 4 Validation of SWGA loci with high intra-species diversity using single template PCR

Gene ID	Species[a]	Strain amplified	π full gene[b]	Amplicon (bp)	π amplicon[b]	Polymorphic positions in unmasked region	Unmasked region (bp)	Sequence differences[c]
PRCDC_0708300	*P. reichenowi*	SY57	0.010899	571	0.008114	4	493	0
PRCDC_0913300	*P. reichenowi*	SY57	0.006656	196	0.014286	1	70	150 bp indel[d]
PF3D7_0915300	*P. gaboni*	SY37	0.012202	213	0.086957	6	69	0
PF3D7_0915300	*P. gaboni*	SY75	0.012202	614	0.025369	12	473	15 bp indel[e]
PRCDC_0931800	*P. reichenowi*	SY57	0.013228	463	0.013274	3	226	0
PRCDC_1208400	*P. reichenowi*	SY57	0.005020	441	0.012821	3	234	0
PF3D7_1209100	*P. gaboni*	SY37	0.010597	385	0.021053	6	285	0
PF3D7_1209100	*P. gaboni*	SY37	0.010597	428	0.012876	3	233	0
PF3D7_1209100	*P. gaboni*	SY75	0.010597	384	0.021053	6	285	0
PF3D7_1417000	*P. gaboni*	SY37	0.010909	391	0.005115	2	391	0
PF3D7_1417000	*P. gaboni*	SY75	0.010909	391	0.005115	2	391	0

[a]For *P. reichenowi*, comparisons were made between PrSY57 and PrCDC; for *P. gaboni*, comparisons were made between PgSY75 and PgSY37.
[b]Repetitive regions that could yield assembly or alignment errors and thus erroneous SNP calls were masked and excluded from the diversity analysis.
[c]Number of sequence differences between SWGA and single template (limiting dilution) PCR derived sequences in both masked and unmasked regions.
[d]Single template PCR identified a 150 bp insertion of repetitive low complexity sequence in a masked region.
[e]Single template PCR identified a 15 bp insertion of repetitive low complexity sequence in a masked region.

Supplementary Table 5 Synteny of *P. gaboni FIKK* family members

FIKK gene ID	Chromosome	Number of syntenic orthologues to the left[a]	Number of syntenic orthologues to the right[b]	Total number of syntenic orthologues[c]	Genomic region of synteny
PGSY75_0102600	1	4	>10	>15	PgSY75_0102200 - PgSY75_0103600+
PGSY75_0301200	3	2	10	13	PgSY75_0301000 - PgSY75_0302200
PGSY75_0424500	4	0	0	1	PgSY75_0424500
PGSY75_0424700	4[d]	0	3	4	PgSY75_0424700 - PgSY75_0452100
PGSY75_0500900	5	0	3	4	PgSY75_0500900 - PgSY75_0501200
PGSY75_0726200	7	1	3	5	PgSY75_0726100 - PgSY75_0726500
PGSY75_0731400	7	0	3	4	PgSY75_0731400 - PgSY75_0731700
PGSY75_0805700	8	10	>10	>21	PgSY75_0804700 - PgSY75_0806700+
PGSY75_0902000	9	3	2	6	PgSY75_0901700 - PgSY75_0902200
PGSY75_0902100	9	4	1	6	PgSY75_0901700 - PgSY75_0902200
PGSY75_0902200	9	5	0	6	PgSY75_0901700 - PgSY75_0902200
PGSY75_0902300	9[d]	0	0	1	PgSY75_0902300
PGSY75_0902400	9[d]	0	0	1	PgSY75_0902400
PGSY75_0902500	9	0	1	2	PgSY75_0902500 - PgSY75_0902600
PGSY75_0902600	9	1	0	2	PgSY75_0902500 - PgSY75_0902600
PGSY75_1016400	10	0	3	4	PgSY75_1016400 - PgSY75_1016800
PGSY75_1039000	10	0	0	1	PgSY75_1039000
PGSY75_1149300	11	0	2	3	PgSY75_1149300 - PgSY75_1149500
PGSY75_1200800	12	1	3	5	PgSY75_1200700 - PgSY75_1201100
PGSY75_1371700	13	0	1	2	PgSY75_1371700 - PgSY75_1371800
PGSY75_1476400	14	2	4	7	PgSY75_1476200 - PgSY75_1476800

[a]All syntenic orthologues of Pf3D7 genes that were located on the contig to the left of the PgSY75 *FIKK* gene.
[b]All syntenic orthologues of Pf3D7 genes that were located on the contig to the right of the PgSY75 *FIKK* gene.
[c]The number is inclusive of *FIKK* genes
[d]For PgSY75 *FIKK* genes that are located on unplaced contigs, the putative chromosomal location was inferred from its respective Pf3D7 orthologue.

Supplementary Table 6 *Laverania* species composition in ape samples

				cytB[d]		*EBA165*[d]		*RH5*[d]		*CyRPA*[d]		*GAPM2*[d]		*FIKK7.2*[d]		*FIKK14*[d]		*FIKK9.15*[d]	
Code[a]	Country[b]	Date[c]	Sample	No. (hap)[e]	Species[f]	No. (hap)[e]	Species[f]	No. (hap)[e]	Species[f]	No. (hap)[e]	Species[f]	No. (hap)[e]	Species[f]	No. (hap)[e]	Species[f]	No. (hap)[e]	Species[f]	No. (hap)[e]	Species[f]
SYpte37	CAM	8/3/12	Blood	8 (1)[g]	C2	3 (1)	C2	3 (1)	C2	2 (1)	C2							4 (1)	C2
SYpte57	CAM	2/13/13	Blood	92 (1)	C1			10 (2)	C1, C2	7 (1)	C1								
SYpte66	CAM	2/12/13	Blood	14 (1)	C2	4 (1)	C2	3 (1)	C2	1 (1)	C2							2 (1)	C2
SYpte70	CAM	11/18/13	RBC[h]	6 (3)	C1, C2					4 (3)	C2							3 (1)	C2
SYpte93	CAM	10/22/13	RBC[h]	11 (1)	C2							2 (1)	C2						
SYpte114	CAM	5/19/14	Blood	3 (1)	C2					12 (2)	C2							5 (1)	C2
SYptt5	CAM	5/7/11	Blood	5 (1)[g]	C2			2 (1)	C2	7 (1)	C2							1 (1)	C2
SYptt7	CAM	4/8/11	Blood	5 (1)	C1			2 (1)	C1	3 (1)	C1								
SYptt13	CAM	4/17/12	Blood	1 (1)	C2	8 (1)	C2	4 (1)	C2	6 (1)	C2								
SYptt15	CAM	7/11/12	Blood	11 (1)[g]	C1			4 (1)	C1	6 (1)	C1								
SYptt20	CAM	2/2/12	Blood	4 (1)[g]	C3			73 (2)	C3	1 (1)	C3	4 (1)	C3						
SYptt45	CAM	10/20/12	Blood	12 (1)	C1					7 (2)	C1	3 (1)	C1						
SYptt47	CAM	10/23/12	Blood	7 (1)	C3			1 (1)	C1										
SYptt61	CAM	2/20/13	Blood	24 (1)	C2	4 (1)	C2	12 (1)	C2	12 (1)	C2	2 (1)	C2					8 (1)	C2
SYptt63	CAM	2/26/13	RBC[h]	11 (1)	C2			2 (2)	C2			1 (1)	C2						
SYptt64	CAM	2/18/13	Blood	8 (1)	C2			1 (1)	C1										
SYptt65	CAM	2/8/13	Blood	14 (1)	C2			3 (2)	C2										
SYptt72	CAM	7/15/13	RBC[h]	6 (2)	C1	8 (1)	C1			1 (1)	C1								
SYptt75	CAM	10/17/13	RBC[h]	15 (3)[g]	C2													8 (1)	C2
SYptt79	CAM	8/27/13	Blood	1 (1)[g]	C3	5 (1)	C3												
SYptt82	CAM	10/28/13	RBC[h]	12 (1)[g]	C2					4 (1)	C2							3 (1)	C2
SYptt117	CAM	5/29/14	Blood	4 (1)	C2					1 (1)	C2	1 (1)	C2					1 (1)	C2
TCptt18	RC	9/8/13	Blood	101(2)	C1	2 (2)	C1	13 (4)	C1, C2										
SAggg3066[i]	CAM	8/19/01	Blood	15 (4)	G1, G2					12 (5)	G2	3 (2)	G2	9 (2)	G2	10 (3)	G1, G2	11 (3)	G2
SAggg3157[i]	CAM	8/31/01	Blood	25 (4)	G1, G2					36 (3)	G1, G2	1 (1)	G1	3 (2)	G1, G2				
BMptt1016	CAM	6/18/05	Faeces	2 (2)	C1, C2	1 (1)	C3			1 (1)	C1								
DGptt540	CAM	9/3/04	Faeces	20 (8)[g]	C1, C3	4 (1)	C1	13 (6)	C1	2 (1)	C1								
GTptt212	RC	3/19/04	Faeces	12 (1)	C1	2 (1)	C1	5 (2)	C1										
GTptt314	RC	10/7/04	Faeces			1 (1)	C2	1 (1)	C2										
GTptt503	RC	2/22/05	Faeces	3 (2)[g]	C3	1 (1)	C3	1 (1)	C3										
GTptt604	RC	4/7/05	Faeces	8 (1)	C2			5 (2)	C2										
GTptt901	RC	2/1/07	Faeces	2 (2)	C2	1 (1)	C1												
GTptt902	RC	2/1/07	Faeces	5 (3)	C1, C3	2 (2)	C1												
LBptt208	CAM	6/7/03	Faeces	4 (4)[g]	C1, C2	1 (1)	C2												
MBptt323	CAM	12/15/03	Faeces	5 (2)	C1	1 (1)	C1												
MTptt157	CAM	10/6/03	Faeces	4 (3)[g]	C2					1 (1)	C2								
NDptt3100	CAR	n/a	Faeces	2 (2)[g]	C2, C3	2 (2)	C3												
BFpts1162	DRC	12/04/06	Faeces	2 (2)	C1	1 (1)	C1												
BFpts1534	DRC	3/1/07	Faeces	1 (1)	C2	1 (1)	C2												
KApts1680	DRC	3/9/07	Faeces	32 (8)[g]	C1, C2	2 (1)	C2	10 (1)	C2	10 (5)	C2							1 (1)	C2
KApts1682	DRC	3/9/07	Faeces	12 (2)	C2	1 (1)	C2												
ONpts1332	DRC	2/8/07	Faeces	1 (1)	C1	1 (1)	C3												
PApts1055	DRC	9/15/06	Faeces	2 (2)	C1, C3	1 (1)	C3												
BQggg60	CAM	2/23/03	Faeces	11 (7)[g]	G1-3			1 (1)	G2										
BQggg61	CAM	2/23/03	Faeces	12 (6)[g]	G1, G2			1 (1)	G2										
BQggg8054	CAM	6/4/12	Faeces	2 (1)	G1	1 (1)	G1												
BYggg3843	CAM	1/17/09	Faeces	3 (1)	G1											1 (1)	G1		

BYggg5024	CAM	11/7/09	Faeces	1 (1)	G1					1 (1)	G1								
CPggg9528	CAM	7/5/13	Faeces	3 (1)	G3	1 (1)	G3												
CPggg9532	CAM	7/5/13	Faeces	3 (1)	G1	1 (1)	G1												
CPggg9664	CAM	7/5/13	Faeces	6 (1)	G3	1 (1)	G3												
DSggg24	CAR	7/1/08	Faeces	10 (2)[g]	G3	2 (2)	G2, G3	1 (1)	G1	1 (1)	G2								
EKggg1182	CAM	8/12/04	Faeces	2 (2)	G2			1 (1)	G2										
GTggg10	RC	2/3/07	Faeces	15 (5)[g]	G1	1 (1)	G2	1 (1)	G1										
GTggg84	RC	2/10/08	Faeces	8 (3)[g]	G1	1 (1)	G1												
GTggg118	RC	6/11/08	Faeces	42 (6)[g]	G1	1 (1)	G1	1 (1)	G1					2 (1)	G1	1 (1)	G1		
GTggg119	RC	5/21/08	Faeces	31 (3)[g]	G3	1 (1)	G3	20 (3)	G1, G3	9 (2)	G2, G3	1 (1)	G3						
GTggg509	RC	3/16/05	Faeces	10 (4)	G1, G2	2 (2)	G2												
GTggg618	RC	4/4/05	Faeces	7 (3)	G2					1 (1)	G3								
GTggg833	RC	9/22/05	Faeces	4 (1)	G3	1 (1)	G3			4 (3)	G2								
LBggg1222	CAM	4/24/05	Faeces	1 (1)[g]	G2	1 (1)	G2												
NDggg3120	CAR	n/a	Faeces	3 (2)[g]	G2			1 (1)	G2										
NDggg3203	CAR	n/a	Faeces	12 (4)[g]	G1-3	1 (1)	G1												

[a]Blood samples were collected opportunistically from individually known chimpanzees housed at the Sanaga Yong Chimpanzee Rescue Center (SY) and the Tchimpounga Chimpanzee Rehabilitation Center (TC). Gorilla blood samples were obtained from bushmeat carcasses of unknown geographic origin (SA) confiscated by the anti-poaching program of the Cameroonian Ministry of Environment and Forestry. Fecal samples were collected from wild-living apes, with their species and subspecies origin determined by mitochondrial DNA analysis (*ptt, P. t. troglodytes; pte, P. t. ellioti; pts, P. t. schweinfurthii; ggg, G. g. gorilla*); a two-letter code indicates the field site of origin as previously reported[1,2].

[b]CAM, Cameroon; RC, Republic of the Congo; DRC, Democratic Republic of the Congo; CAR, Central African Republic.

[c]Date listed as mm/dd/yy

[d]Single template amplified loci of *Laverania* mitochondrial (*cytB*) and nuclear (*EBA165, RH5, CyRPA*, *GAPM2*, *FIKK7.2*, *FIKK14*, and *FIKK9.15*) genes.

[e]No., number of single template PCR-derived sequences, with brackets indicating the number of distinguishable haplotypes (hap). See Supplementary Table 8 for GenBank accession numbers.

[f]Ape *Laverania* species present in sample: C1, *P. reichenowi*; C2, *P. gaboni*; C3, *P. billcollinsi*; G1, *P. praefalciparum*; G2, *P. alderi*; G3, *P. blacklocki.*

[g]These sequences have previously been reported[1,3].

[h]RBC, red blood cells were purified by Lymphoprep density gradient centrifugation.

[i]Gene sequences were amplified from SWGA pre-treated sample DNA.

Supplementary Table 7 Syntenic orthologues of *P. falciparum* and *P. reichenowi DNAJ* and *PHISTb* genes in *P. gaboni*

Pf3D7 ID	Orthologue in PrCDC	Orthologue in PgSY75	Family	Gene Product Description
PF3D7_0114000	PRCDC_0112200	PGSY75_0114000	*DNAJ*	exported protein family 1 (EPF1)
PF3D7_0220400	PRCDC_0219300	PGSY75_0220400	*DNAJ*	DNAJ protein, putative, pseudogene
PF3D7_0500800	PRCDC_0500100	NF[a]	*DNAJ*	mature parasite-infected erythrocyte surface antigen,erythrocyte membrane protein 2 (MESA)
PF3D7_0523400	PRCDC_0522500	PGSY75_0523400	*DNAJ*	DNAJ protein, putative
PF3D7_1039100	NF	NF	*DNAJ*	DNAJ protein, putative, pseudogene
PF3D7_1102200	PRCDC_1100700	PGSY75_1102200	*DNAJ*	DNAJ protein, putative
PF3D7_1149600	PRCDC_1147500	bin[b]	*DNAJ*	DNAJ protein, putative
PF3D7_1253000	PRCDC_1252600	bin	*DNAJ*	gametocyte erythrocyte cytosolic protein (GECO)
PF3D7_1401100	PRCDC_1400400	PGSY75_1401100	*DNAJ*	DNAJ protein, putative
PF3D7_0201600	PRCDC_0200700	PGSY75_0201600	*PHISTb*	*Plasmodium* exported protein (PHISTb), unknown function
PF3D7_0401800	PRCDC_0004900	bin	*PHISTb*	*Plasmodium* exported protein (PHISTb), unknown function (PfD80)
PF3D7_0402100	PRCDC_0005200	PGSY75_0402100	*PHISTb*	*Plasmodium* exported protein (PHISTb), unknown function
PF3D7_0424600	PRCDC_0421800	PGSY75_0424600	*PHISTb*	*Plasmodium* exported protein (PHISTb), unknown function
PF3D7_0424800	PRCDC_0422000	PGSY75_0424800	*PHISTb*	*Plasmodium* exported protein (PHISTb), unknown function
PF3D7_0532300	PRCDC_0531300	PGSY75_0532300	*PHISTb*	*Plasmodium* exported protein (PHISTb), unknown function
PF3D7_0532400	PRCDC_0531400	bin	*PHISTb*	lysine-rich membrane-associated PHISTb protein (LyMP)
PF3D7_0601500	PRCDC_0600100	NF	*PHISTb*	*Plasmodium* exported protein (PHISTb), unknown function
PF3D7_0631100	PRCDC_0629500	PGSY75_0631100	*PHISTb*	*Plasmodium* exported protein (PHISTb), unknown function
PF3D7_0702100	PRCDC_0700300	PGSY75_0702100	*PHISTb*	*Plasmodium* exported protein (PHISTb), unknown function, pseudogene
PF3D7_0731300	PRCDC_0728100	NF	*PHISTb*	*Plasmodium* exported protein (PHISTb), unknown function (PfG174)
PF3D7_0831000	PRCDC_0830300	PGSY75_0831000	*PHISTb*	*Plasmodium* exported protein (PHISTb), unknown function (GEXP09)
PF3D7_0902700	NF	PGSY75_0902700	*PHISTb*	*Plasmodium* exported protein (PHISTb), unknown function, pseudogene
PF3D7_0936900	PRCDC_0935000	PGSY75_0936900	*PHISTb*	*Plasmodium* exported protein (PHISTb), unknown function, pseudogene
PF3D7_0937000	PRCDC_0935100	PGSY75_0937000	*PHISTb*	*Plasmodium* exported protein (PHISTb), unknown function
PF3D7_1102500	PRCDC_1101000	PGSY75_1102500	*PHISTb*	*Plasmodium* exported protein (PHISTb), unknown function (GEXP02)
PF3D7_1201000	PRCDC_1200400	PGSY75_1201000	*PHISTb*	*Plasmodium* exported protein (PHISTb), unknown function
PF3D7_1252700	PRCDC_1252100	PGSY75_1252700	*PHISTb*	*Plasmodium* exported protein (PHISTb), unknown function
PF3D7_1252800	PRCDC_1252200	PGSY75_1252800	*PHISTb*	*Plasmodium* exported protein (PHISTb), unknown function
PF3D7_1372100	PRCDC_1370000	PGSY75_1372100	*PHISTb*	*Plasmodium* exported protein (PHISTb), unknown function (GEXP04)
PF3D7_1401600	PRCDC_1400900	bin	*PHISTb*	*Plasmodium* exported protein (PHISTb), unknown function
PF3D7_1476200	PRCDC_0060600	PGSY75_1476200	*PHISTb*	*Plasmodium* exported protein (PHISTb), unknown function
PF3D7_1476300	PRCDC_1475300	PGSY75_1476300	*PHISTb*	*Plasmodium* exported protein (PHISTb), unknown function

PF3D7_1477500	PRCDC_1476500	bin	*PHISTb*	*Plasmodium* exported protein (PHISTb), unknown function
PF3D7_0102200	PRCDC_0100100	PGSY75_0102200	*PHISTb DNAJ*	Ring-infected erythrocyte surface antigen (RESA)
PF3D7_0201700	PRCDC_0200800	PGSY75_0201700	*PHISTb DNAJ*	DNAJ protein, putative
PF3D7_0220100	PRCDC_0219000	PGSY75_0220100	*PHISTb DNAJ*	DNAJ protein, putative
PF3D7_1038800	PRCDC_1037800	PGSY75_1038800	*PHISTb DNAJ*	RESA-like protein with PHIST and DNAJ domains
PF3D7_1149200	PRCDC_1147100	bin	*PHISTb DNAJ*	Ring-infected erythrocyte surface antigen
PF3D7_1149500	PRCDC_1147400	PGSY75_1149500	*PHISTb DNAJ*	Ring-infected erythrocyte surface antigen 2, pseudogene (RESA2)
PF3D7_1201100	PRCDC_1200500	PGSY75_1201100	*PHISTb DNAJ*	RESA-like protein with PHIST and DNAJ domains

[a]NF, not found in assembly.
[b]bin, orthologue in unplaced bin.

Supplementary Table 8 GenBank accession codes for single template amplified ape *Laverania* gene sequences

Sample	*cytb*	Accession No.	*EBA165*	Accession No.	*RH5*	Accession No.	*CyRPA*	Accession No.	*GAPM2*	Accession No.	*FIKK7.2*	Accession No.	*FIKK14*	Accession No.	*FIKK9.15*	Accession No.
**SYpte37**	SYpte37_100.1	=HM234979	SYpte37_20.1	KT824360	SYpte37_5.1	KT824408	SYpte37_20.1	KT824271							SYpte37_20.1	=SYptt75_60.1
**SYpte57**	SYpte57_4000.4	KT824311			SYpte57_60.1	KT824409	SYpte57_60.2	KT824272								
					SYpte57_60.12	=SYpte37_5.1										
**SYpte66**	SYpte66_20.1	=HM234979	SYpte66_20.2	=SYpte37_20.1	SYpte66_5.1	=SYpte37_5.1	SYpte66_60.6	=SYpte37_20.1							SYpte66_60.3	=SYptt75_60.1
**SYpte70**	SYpte70_6000.10	=HM234979					SYpte70_50.1	=KApts1680_60.1							SYpte70_60.1	=SYptt75_60.1
	SYpte70_6000.1	=HM235098					SYpte70_30.11	=SYpte114_30.1								
	SYpte70_6000.2	KT824306					SYpte70_30.8	=SYptt61_60.1								
**SYpte93**	SYpte93_60.2	=HM234979							SYpte93_60.2	KU193800						
**SYpte114**	SYpte114_5.4	=HM235098					SYpte114_30.1	KT824270							SYpte114_60.1	=SYptt75_60.1
							SYpte114_30.5	=SYptt13_100.2								
**SYptt5**	SYptt5_200.2	=HM234979			SYptt5_5.1	KT824415	SYptt5_100.1	KT824277							SYptt5_60.1	=SYptt75_60.1
**SYptt7**	SYptt7_200.1	KT824309			SYptt7_60.12	KT824421	SYptt7_60.6	KT824280								
**SYptt13**	SYptt13_6000.11	=HM234979	SYptt13_20.1	KT824365	SYptt13_5.1	KT824410	SYptt13_100.2	KT824273								
**SYptt15**	SYptt15_20.1	=HM234980			SYptt15_60.5	KT824411	SYptt15_20.1	KT824274								
**SYptt20**	SYptt13_6000.12	=HM234993			SYptt20_20.40	KT824412	SYptt20_40.6	KT824275	SYptt20_400.3	KU193798						
					SYptt20_5.1	KT824413										
**SYptt45**	SYptt45_20.1	KT824310					SYptt45_500.1	KT824276	SYptt45_80000.10	KU193799						
							SYptt45_500.4	=SYpte57_60.2								
**SYptt47**	SYptt47_20.1	=HM234993			SYptt47_10.5	KT824414										
**SYptt61**	SYptt61_20.1	=HM234979	SYptt61_5.3	=SYptt13_20.1	SYptt61_5.4	KT824416	SYptt61_60.1	KT824278	SYptt61_2400.1	KU193797					SYptt61_60.2	KT824384
**SYptt63**	SYptt63_20.1	KT824312			SYptt63_5.4	KT824417			SYptt63_2500.6	KU193802						
					SYptt63_60.10	KT824418										
**SYptt64**	SYptt64_40.6	=SYptt117_5.1			SYptt64_60.5	KT824419										
**SYptt65**	SYptt65_20.1	=SYptt117_5.1			SYptt65_6.9	KT824420										
					SYptt65_60.1	=SYpte37_5.1										
**SYptt72**	SYptt72_12000.1	KT824307	SYptt72_5.1	=MBptt323_5.4			SYptt72_100.6	KT824279								
	SYptt72_3000.8	KT824308														
**SYptt75**	SYptt75_6000.4	=HM234979													SYptt75_60.1	KT824385
	SYptt75_1000.4	KU302812														
	SYptt75_1000.6	=SYptt117_5.1														
**SYptt79**	SYptt79_60.3	=HM234993	SYptt79_5.1	KT824371												
**SYptt82**	SYptt82_60.1	=HM234979					SYptt82_30.2	=SYpte37_20.1							SYptt82_60.8	=SYptt75_60.1
**SYptt117**	SYptt117_5.1	KT824304					SYptt117_60.1	=SYptt13_100.2	SYptt117_60.7	KU193801					SYptt117_60.3	=SYptt75_60.1
**TCptt18**	TCptt18_3000.38	=HM234980	TCptt18_500.2	KT824374	TCptt18_20.6	KT824422										
	TCptt18_30000.4	=SYptt7_200.1	TCptt18_500.10	KT824373	TCptt18_5.8	KT824423										
					TCptt18_60.2	KT824425										
					TCptt18_60.11	KT824424										
**SAggg3066**	SAggg3066_1000.1	=HM234986					SAggg3066_60.1	KT824253	SAggg3066_200.2	KU193803	SAggg3066_60.1	KT824375	SAggg3066_60.1	KT824387	SAggg3066_100.1	KT824380
	SAggg3066_100.9	=HM235017					SAggg3066_60.2	KT824254	SAggg3066_200.5	KU193804	SAggg3066_60.5	KT824376	SAggg3066_60.2	KT824388	SAggg3066_100.2	KT824381
	SAggg3066_1000.6	KT824302					SAggg3066_60.3	KT824255					SAggg3066_60.4	=GTggg118_10.10	SAggg3066_100.6	KT824382
	SAggg3066_100.8	=HM235041					SAggg3066_60.5	KT824256								
							SAggg3066_60.9	KT824257								
**SAggg3157**	SAggg3157_30.35	=HM234988					SAggg3157_60.22	KT824258	SAggg3157_1000.12	KU193795	SAggg3157_60.21	KT824377				
	SAggg3157_30.30	=HM234986					SAggg3157_60.28	KT824259			SAggg3157_60.23	KT824378				
	SAggg3157_30.29	KT824303					SAggg3157_60.1	=BYggg5024_5.6								
	SAggg3157_30.38	=HM235041														
**BMptt1016**	BMptt1016_120.10	=HM234979	BMptt1016_5.3	KT824322			BMptt1016_5.11	=SYptt7_60.6								
	BMptt1016_120.7	=HM234980														
**DGptt540**			DGptt540_40.1	KT824327	DGptt540_30.1	KT824392	DGptt540_60.1	KT824260								

					DGptt540_30.3	KT824393										
					DGptt540_30.10	KT824391										
					DGptt540_5.5	KT824394										
					DGptt540_30.4	=SYptt64_60.5										
					DGptt540_5.1	=SYptt15_60.5										
**GTptt212**	GTptt212_10.1	=HM235029	GTptt212_5.6	=GTptt902_5.1	GTptt212_5.1	KT824401										
					GTptt212_5.4	KT824402										
**GTptt314**			GTptt314_5.6	KT824337	GTptt314_5.4	KT824403										
**GTptt503**			GTptt503_5.2	KT824338	GTptt503_5.6	KT824404										
**GTptt604**	GTptt604_10.1	=HM234979			GTptt604_5.1	KT824405										
					GTptt604_5.3	KT824406										
**GTptt901**	GTptt901_5.1	=HM234979	GTptt901_5.3	=MBptt323_5.4												
	GTptt901_30.9	=HM235112														
**GTptt902**	GTptt902_30.1	=SYptt7_200.1	GTptt902_5.8	KT824342												
	GTptt902_30.2	=HM235018	GTptt902_5.1	KT824341												
	GTptt902_30.8	KT824294														
**LBptt208**			LBptt208_5.1	KT824350												
**MBptt323**	MBptt323_5.2	KT824297	MBptt323_5.4	KT824352												
	MBptt323_5.4	KT824298														
**MTptt157**							MTptt157_5.4	=SYptt5_100.1								
**NDptt3100**			NDptt3100_5.11	KT824356												
			NDptt3100_5.8	KT824357												
**BFpts1162**	BFpts1162_5.3	KT824282	BFpts1162_5.5	KT824316												
	BFpts1162_5.1	=ONpts1332_5.4														
**BFpts1534**	BFpts1534_5.1	KT824286	BFpts1534_5.1	KT824319												
**KApts1680**			KApts1680_60.12	=BF1534_5.1	KApts1680_30.1	KT824407	KApts1680_3.1	KT824265							KApts1680_100.1	KT824383
							KApts1680_60.2	KT824268								
							KApts1680_60.3	KT824269								
							KApts1680_60.10	KT824267								
							KApts1680_60.1	KT824266								
**KApts1682**	KApts1682_120.1	=HM235050	KApts1682_60.9	KT824344												
	KApts1682_120.5	=HM235052														
**ONpts1332**	ONpts1332_10.4	KT824299	ONpts1332_5.1	KT824358												
**PApts1055**	PApts1055_5.2	KT824300	PApts1055_10.10	KT824359												
	PApts1055_5.4	KT824301														
**BQggg60**					BQggg60_6.11	KT824390										
**BQggg61**					BQggg61_6.11	=BQggg60_6.11										
**BQggg8054**	BQggg8054_30.1	=HM235041	BQggg8054_5.9	KT824323												
**BYggg3843**	BYggg3843_5.1	=HM235041											BYggg3843_10.11	KT824386		
**BYggg5024**	BYggg5024_5.1	KT824288					BYggg5024_5.6	KT824252								
**CPggg9528**	CPggg9528_10.11	=HM235064	CPggg9528_5.5	KT824325												
**CPggg9532**	CPggg9532_10.3	KT824289	CPggg9532_5.1	KT824326												
**CPggg9664**	CPggg9664_10.1	=HM235064	CPggg9664_5.10	=CPggg9528_5.5												
**DSggg24**			DSggg24_10.3	KT824328	DSggg24_6.7	KT824395	DSggg24_5.11	KT824261								
			DSggg24_10.11	=GTggg119_5.5												
**EKggg1182**	EKggg1182_10.4	KT824290			EKggg1182_8.8	KT824396										
	EKggg1182_10.9	=HM234987														
**GTggg10**			GTggg10_10.8	KT824329	GTggg10_6.4	KT824397										
**GTggg84**			GTggg84_5.3	KT824334												
**GTggg118**	GTggg118_10.10	KT824292	GTggg118_5.8	KT824330	GTggg118_10.4	KT824398					GTggg118_10.8	KT824379	GTggg118_10.10	KT824389		
**GTggg119**			GTggg119_5.5	KT824331	GTggg119_10.3	KT824399	GTggg119_4.8	KT824262	GTggg119_5.2	KU193796						
					GTggg119_5.8	KT824400	GTggg119_5.2	=DSggg24_5.11								
					GTggg119_10.1	=DSggg24_6.7										

**GTggg509**	GTggg509_10.2	=HM234988	GTggg509_5.6	KT824333				
	GTggg509_10.5	KT824293	GTggg509_10.10	KT824332				
	GTggg509_10.10	=HM234987						
	GTggg509_10.11	=HM234986						
**GTggg618**	GTggg618_10.6	=HM235042					GTggg618_5.9	=GTggg119_4.8
	GTggg618_10.7	KT824291						
	GTggg618_10.8	=HM234986						
**GTggg833**	GTggg833_30.4	=HM235064	GTggg833_5.4	=CPggg9528_5.5			GTggg833_5.2	KT824263
							GTggg833_5.4	KT824264
							GTggg833_5.1	=DSggg24_5.11
**LBggg1222**			LBggg1222_5.5	KT824349				
**NDggg3120**					NDggg3120_6.11	=BQggg60_6.11		
**NDggg3203**			NDggg3203_10.3	KT824355				

Supplementary Table 9 Illumina sequencing runs and accession codes

Sample	Sample Name	Library Preparation Kit[a]	I7 Index[b]	I7 Index Sequence[b]	SRA Accession Number
SY37	SY37_1	TruSeq LT	A002	CGATGT	SRR2414471
SY37	SY37_2	TruSeq LT	A004	TGACCA	SRR2414472
SY37	SY37_3	TruSeq LT	A005	ACAGTG	SRR2414483
SY37	SY37_4	TruSeq LT	A006	GCCAAT	SRR2414487
SY37	SY37_5	TruSeq LT	A007	CAGATC	SRR2414488
SY37	SY37_6	TruSeq LT	A013	AGTCAA	SRR2414489
SY37	SY37_7	TruSeq LT	A014	AGTTCC	SRR2414490
SY37	SY37_8	TruSeq LT	A015	ATGTCA	SRR2414491
SY37	SY37_9	TruSeq LT	A016	CCGTCC	SRR2414492
SY37	SY37_10	TruSeq LT	A012	CTTGTA	SRR2414493
SY37	SY37_11	TruSeq LT	A005	ACAGTG	SRR2414473
SY37	SY37_12	TruSeq LT	A006	GCCAAT	SRR2414474
SY75	SY75_1	TruSeq LT	A015	ATGTCA	SRR2414475
SY75	SY75_2	TruSeq LT	A015	ATGTCA	SRR2414476
SY75	SY75_5kb_1	Nextera Mate Pair	A005	ACAGTG	SRR2414477
SY75	SY75_8kb_1	Nextera Mate Pair	A012	CTTGTA	SRR2414478
SY75	SY75_3kb_1	Nextera Mate Pair	A006	GCCAAT	SRR2414479
SY75	SY75_5kb_2	Nextera Mate Pair	A018	GTCCGC	SRR2414480
SY75	SY75_9kb_1	Nextera Mate Pair	A019	GTGAAA	SRR2414481
SY75	SY75_3kb_2	Nextera Mate Pair	A006	GCCAAT	SRR2414482
SY57	SY57_1	TruSeq LT	A005	ACAGTG	SRR2414484
SY57	SY57_2	TruSeq LT	A006	GCCAAT	SRR2414485
SY57	SY57_3	TruSeq LT	A015	ATGTCA	SRR2414486

[a]Library preparation used for sample.
[b]Index IDs and sequences used for sequencing run multiplexing.

Supplementary References

1. Liu W*, et al.* Origin of the human malaria parasite *Plasmodium falciparum* in gorillas. *Nature* **467**, 420-425 (2010).
2. Liu W*, et al.* African origin of the malaria parasite *Plasmodium vivax*. *Nat. Commun.* **5**, 3346 (2014).
3. Larremore DB*, et al.* Ape parasite origins of human malaria virulence genes. *Nat. Commun.* **6**, 8368 (2015).
4. Guindon S, Delsuc F, Dufayard JF, Gascuel O. Estimating maximum likelihood phylogenies with PhyML. *Methods Mol. Biol.* **537**, 113-137 (2009).
5. Felsenstein J. Confidence limits on phylogenies: an approach using the bootstrap. *Evolution* **39**, 783-791 (1985).
6. Smith JM. Analyzing the mosaic structure of genes. *J. Mol. Evol.* **34**, 126-129 (1992).
